# Nature-inspired platform nanotechnology for RNA delivery to myeloid cells and their bone marrow progenitors

**DOI:** 10.1038/s41565-024-01847-3

**Published:** 2025-02-03

**Authors:** Stijn R. J. Hofstraat, Tom Anbergen, Robby Zwolsman, Jeroen Deckers, Yuri van Elsas, Mirre M. Trines, Iris Versteeg, Daniek Hoorn, Gijs W. B. Ros, Branca M. Bartelet, Merel M. A. Hendrikx, Youssef B. Darwish, Teun Kleuskens, Francisca Borges, Rianne J. F. Maas, Lars M. Verhalle, Willem Tielemans, Pieter Vader, Olivier G. de Jong, Tommaso Tabaglio, Dave Keng Boon Wee, Abraham J. P. Teunissen, Eliane Brechbühl, Henk M. Janssen, P. Michel Fransen, Anne de Dreu, David P. Schrijver, Bram Priem, Yohana C. Toner, Thijs J. Beldman, Mihai G. Netea, Willem J. M. Mulder, Ewelina Kluza, Roy van der Meel

**Affiliations:** 1 Laboratory of Chemical Biology, Department of Biomedical Engineering, Eindhoven University of Technology, Eindhoven, the Netherlands; 2 Institute for Complex Molecular Systems (ICMS), Eindhoven University of Technology, Eindhoven, the Netherlands; 3 Department of Internal Medicine and Radboud Center for Infectious Diseases (RCI), Radboud University Medical Center, Nijmegen, the Netherlands; 4 Biotrip B.V., Eindhoven, the Netherlands; 5 CDL Research & Department of Experimental Cardiology, University Medical Center Utrecht, Utrecht, the Netherlands; 6 Department of Pharmaceutics, Utrecht Institute of Pharmaceutical Sciences (UIPS), Utrecht University, Utrecht, the Netherlands; 7 Institute of Molecular and Cell Biology (IMCB), Agency for Science, Technology and Research (A*STAR), Singapore, Singapore; 8 BioMedical Engineering and Imaging Institute, Icahn School of Medicine at Mount Sinai, New York, NY USA; 9 Department of Diagnostic, Molecular, and Interventional Radiology, Icahn School of Medicine at Mount Sinai, New York, NY USA; 10 Cardiovascular Research Institute, Icahn School of Medicine at Mount Sinai, New York, NY USA; 11 Icahn Genomics Institute, Icahn School of Medicine at Mount Sinai, New York, NY USA; 12 SyMO-Chem B.V., Eindhoven, the Netherlands; 13 Department of Immunology and Metabolism, Life and Medical Sciences Institute, University of Bonn, Bonn, Germany

**Keywords:** Nanoparticles, Drug delivery

## Abstract

Nucleic acid therapeutics are used for silencing, expressing or editing genes in vivo. However, their systemic stability and targeted delivery to bone marrow resident cells remains a challenge. In this study we present a nanotechnology platform based on natural lipoproteins, designed for delivering small interfering RNA (siRNA), antisense oligonucleotides and messenger RNA to myeloid cells and haematopoietic stem and progenitor cells in the bone marrow. We developed a prototype apolipoprotein nanoparticle (aNP) that stably incorporates siRNA into its core. We then created a comprehensive library of aNP formulations and extensively characterized their physicochemical properties and in vitro performance. From this library, we selected eight representative aNP-siRNA formulations and evaluated their ability to silence lysosomal-associated membrane protein 1 (*Lamp1*) expression in immune cell subsets in mice after intravenous administration. Using the most effective aNP identified from the screening process, we tested the platform’s potential for therapeutic gene silencing in a syngeneic murine tumour model. We also demonstrated the aNP platform’s suitability for splice-switching with antisense oligonucleotides and for protein production with messenger RNA by myeloid progenitor cells in the bone marrow. Our data indicate that the aNP platform holds translational potential for delivering various types of nucleic acid therapeutics to myeloid cells and their progenitors.

## Abstract (web summary)

In this study, the authors present optimization and efficacy testing of apolipoprotein-based lipid nanoparticles for delivering various nucleic acid therapeutics in vivo to immune cells and their progenitors in the bone marrow.

## Main

Genetic drugs’ real-life potential is critically dependent on delivery technologies that prevent the nucleic acid payload’s premature degradation while actively delivering it to the cells of interest^1^. In past decades, lipid nanoparticle (LNP) technology emerged that enabled the first small interfering RNA (siRNA) therapeutic’s clinical translation, involving gene silencing in hepatocytes to treat hereditary transthyretin amyloidosis^2,3^. Furthermore, LNP technology was essential for the COVID-19 messenger RNA (mRNA) vaccines^4,5^, and facilitates in vivo gene editing approaches currently undergoing clinical evaluation^6,7^. To enable targeting of tissues and cells beyond the liver^8^, several LNP modification and screening strategies have been developed and evaluated preclinically. These include incorporating charged phospholipids^9,10^, antibody surface modification of LNPs^11–16^ and DNA barcoding strategies^17–19^. However, platform technologies that possess biocompatible and tunable biodistribution features must be developed to unlock RNA therapeutics’ full potential for systemic delivery.

**Fig. 1:**
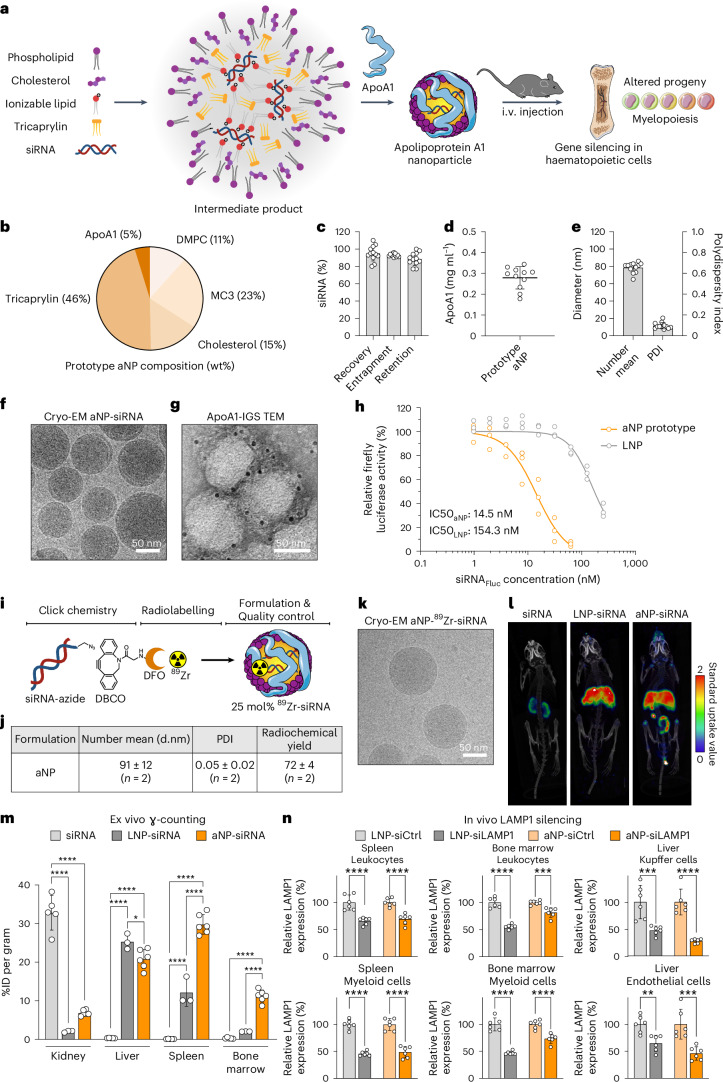
Prototyping aNP platform technology for siRNA delivery to the myeloid cell compartment. **a**, Schematic representation of siRNA apolipoprotein nanoparticle (aNP-siRNA) platform technology. **b**, Prototype aNP composition (wt%). **c**, siRNA recovery, entrapment and retention (*n* = 12 formulation batches). **d**, Apolipoprotein A1 (apoA1) content (*n* = 11 formulation batches). **e**, Hydrodynamic diameter presented as the number mean average, and the associated dispersity (*n* = 12 formulation batches). **f**, Cryo-EM. **g**, Transmission electron microscopy and apoA1-immunogold staining (IGS). **h**, Firefly luciferase (Fluc) reporter gene silencing in RAW 264.7 cells stably expressing Fluc and *Renilla* luciferase of prototype aNP compared with LNPs containing Fluc siRNA (*n* = 3 formulation batches). **i**, Complexation of zirconium-89 (^89^Zr) to DFO-siRNA. **j**, aNP-^89^Zr-siRNA physicochemical analysis (*n* = 2 formulation batches). d.nm, diameter (nm). **k**, aNP-^89^Zr-siRNA cryo-EM analysis. **l**, Positron emission tomography–computed tomography (PET–CT) imaging of mice 24 h after intravenous administration of unformulated ^89^Zr-siRNA, LNP-^89^Zr-siRNA and aNP-^89^Zr-siRNA. **m**, Quantitative biodistribution of unformulated ^89^Zr-siRNA, LNP-^89^Zr-siRNA and aNP-^89^Zr-siRNA 24 h after intravenous administration in mice (8 µCi per mouse) as determined by ex vivo gamma counting. Radioactivity is expressed as the percentage injected dose per gram of tissue (%ID per gram). Data represent mean ± s.d. of one experiment, each data point represents one animal (*n* = 3, 5 or 6 mice). The data were analysed by one-way ANOVA with a Bonferroni post-hoc test. **n**, Functional gene silencing of lysosomal-associated membrane protein 1 (*Lamp1*) expression in liver Kupffer cells and endothelial cells, as well as splenic and bone marrow leukocytes and myeloid cells following intravenous administration (4 × 0.5 mg kg^–1^ in six days, readout 36 h after the last injection) of LNP or aNP containing siCtrl or siLAMP1, as determined by flow cytometry. Data represent mean ± s.d. of one experiment (*n* = 6 mice) and analysed by one-way ANOVA using a Bonferroni post-hoc test. *P ≤ 0.05, ***P ≤ 0.001, ****P ≤ 0.0001. Source data

Here we introduce a nanodelivery strategy based on lipoprotein trafficking, designed for extrahepatic delivery to immune cells. Lipoproteins are endogenous, nanosized transport systems composed of apolipoproteins and aggregates of fatty molecules^20^. They inherently interact with various cells and exhibit compelling features for RNA delivery^21^. The widely studied apolipoprotein A1 (apoA1) is high-density lipoprotein’s (HDL) main protein constituent^22^. Capitalizing on apoA1’s natural function, we developed platform nanotechnology^23,24^ to deliver RNA to myeloid cells and haematopoietic stem and progenitor cells (HSPCs) in the bone marrow^25–27^ (Fig. 1a). After establishing a prototype apolipoprotein nanoparticle (aNP) that stably incorporates siRNA in its core, we established a comprehensive aNP-siRNA library and characterized the in vitro properties of individual formulations. We subsequently selected eight aNP-siRNA formulations that are representative of the library’s diversity, and in vivo screened their capacity to silence lysosomal-associated membrane protein 1 (*Lamp1*) expression in diverse immune cell subsets in mice. We also studied a lead aNP-siRNA candidate’s physicochemical properties, tissue biodistribution and its potential for therapeutic gene silencing in a syngeneic murine tumour model. Finally, we demonstrate the aNP platform’s suitability for splice-switching with antisense oligonucleotides (ASOs) and for in vivo protein production with mRNA by myeloid progenitor cells in the bone marrow.

## Prototyping aNPs for siRNA delivery

**Extended Data Fig. 1:**
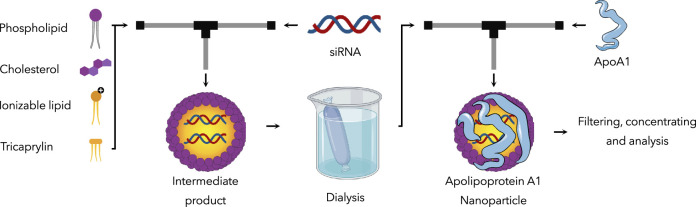
aNP-siRNA formulation procedure. Lipid molecules dissolved in ethanol are rapidly mixed via a T-junction with siRNA dissolved in sodium acetate buffer (25 mM, pH 4). The resulting intermediate product is then dialysed against PBS buffer (pH 7.4). Next, the intermediate product is rapidly mixed with apoA1 dissolved in PBS. The final product is filtered, concentrated, and analyzed.

First, we developed a two-step flow manufacturing process for an aNP prototype containing siRNA using a T-junction mixer^28^ (Extended Data Fig. 1). The prototype aNP components depicted in Fig. 1b include 1,2-dimyristoyl-*sn*-glycero-3-phosphocholine (DMPC), cholesterol, tricaprylin, a triglyceride that makes up the core matrix accomodating siRNA complexed with the ionizable lipid (6Z,9Z,28Z,31Z)-heptatriacont-6,9,28,31-tetraene-19-yl-4-(dimethylamino)butanoate (MC3)^3,29,30^, and apoA1. Importantly, we found siRNA recovery, entrapment and retention to be higher than 80% (Fig. 1c), which is indicative of efficient siRNA incorporation. The prototype aNPs contained consistent quantities of apoA1 (Fig. 1d), and had a mean hydrodynamic diameter of approximately 80 nm (Fig. 1e), with a size dispersity of 0.1, as measured by dynamic light scattering (DLS). We qualitatively corroborated both size and size distribution by cryogenic electron microscopy (cryo-EM, Fig. 1f), which revealed spherical core–shell structures. Using a gold nanoparticle antibody-labelling method, we visualized apoA1’s integration in the aNP by negative staining transmission electron microscopy (TEM, Fig. 1g).

**Extended Data Fig. 2:**
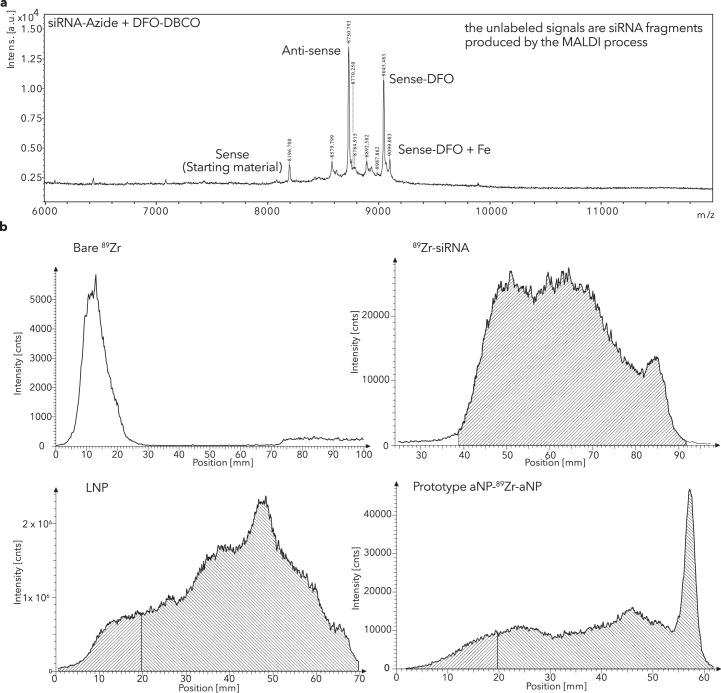
Radio-thin layer chromatography (radio-TLC) of aNP-^89^Zr-siRNA. **a**. MALDI spectra indicating conjugation of DFO to the siRNA sense strand **b**. Radio-TLC analysis of free ^89^Zr (*top left*) showing a peak at 15 mm. The peak shifts when ^89^Zr is chelated to DFO-siRNA to yield ^89^Zr-siRNA (*top right*). ^89^Zr-siRNA was used for formulation of the prototype aNP (*bottom right*) and a control LNP (*bottom left*).

In an in vitro silencing assay using RAW 264.7 murine macrophages that stably express firefly- and *Renilla* luciferase, we determined an IC_50_ value of 14.5 nM (Fig. 1h), which was considerably better than 154.3 nM for a clinically approved MC3-based LNP control formulation (Supplementary Table 1). We then developed a unique siRNA radiolabelling strategy (Fig. 1i and Extended Data Fig. 2) to quantitatively measure siRNA biodistribution by in vivo positron emission tomography (PET) and ex vivo gamma counting. Using azide–alkyne click chemistry, we functionalized siRNA with desferrioxamine B (DFO) to enable radiolabelling with zirconium-89 (^89^Zr; Fig. 1i and Extended Data Fig. 2). Although DLS disclosed a slightly increased hydrodynamic diameter of approximately 91 nm with a uniform size distribution (Fig. 1j), cryo-EM (Fig. 1k) showed the same spherical core–shell structure as the non-labelled aNP (Fig. 1f). In addition to unformulated ^89^Zr-labelled siRNA (^89^Zr-siRNA), we intravenously administered LNP-^89^Zr-siRNA and aNP-^89^Zr-siRNA to mice (Fig. 1l,m). After 24 h, PET imaging uncovered unformulated ^89^Zr-siRNA to be renally cleared (Fig. 1l), whereas aNP-formulated ^89^Zr-siRNA accumulated much stronger in the spleen and bone marrow as compared with LNP delivery. Quantitative ex vivo gamma counting corroborated these in vivo PET imaging findings (Fig. 1m).

**Extended Data Fig. 3:**
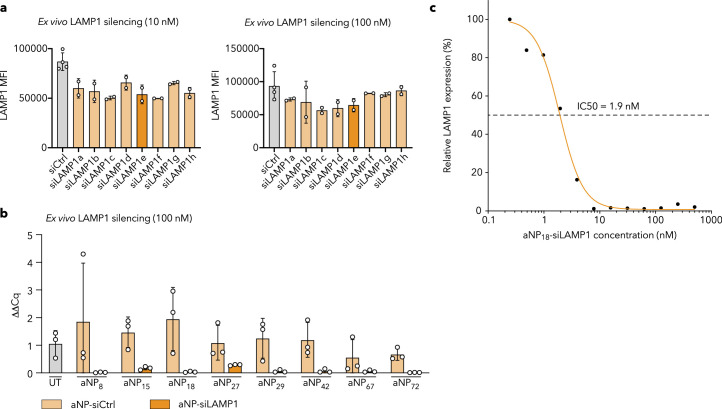
*Ex vivo* LAMP1 silencing in bone marrow-derived macrophages. **a**. *Ex vivo* screening of siLAMP1 constructs (Supplementary Table 4) using bone marrow-derived macrophages (BMDMs). Cells were transfected with siRNA formulated with Lipofectamine RNAiMAX at a concentration of 10 and 100 nM for 24 h. 72 h after transfections, cells were harvested and prepared for flow cytometry. LAMP1 mean fluorescence intensity was measured by flow cytometry. For further experiments, siLAMP1e was selected (dark orange bar). Data represents mean ± SD of one experiment (minimal of n = 2 biological replicates). **b**. *Ex vivo* LAMP1 silencing in BMDMs after transfection with aNP-siLAMP1. Cells were incubated for 24 h with aNP-siRNAs. 48 h after incubation, the cells were processed for RT-qPCR. *Lamp1* expression levels were determined by RT-qPCR and the ΔΔCq-values are displayed. The primers are listed in Supplementary Table 3. Data represent mean ± SD of one experiment (n = 3 donors). **c**. *Ex vivo Lamp1* silencing in bone marrow-derived macrophages aNP_18_ dose response. *Lamp1* expression was determined 48 h after incubation with RT-qPCR and the ΔCq-expression normalized to siCtrl-aNP_18_ at an equivalent concentration. The % knockdown is calculated according to the following formula: %KD = (1 − ∆∆Cq) × 100. The absolute IC50 value is 1.9 nM. Source data

We next evaluated the prototype aNP’s silencing potential in leukocytes. To achieve this, we first ex vivo-screened eight siRNA constructs targeting *Lamp1* mRNA (Extended Data Fig. 3a and Supplementary Table 4). LAMP1 is expressed on the cell surface and can be quantified by flow cytometry^18^. We formulated the most potent anti-LAMP1 siRNA (siLAMP1) into the prototype aNP formulation and administered four intravenous aNP-siLAMP1 doses (0.5 mg kg^–1^ siLAMP1, injected 36 h apart) to mice. Aside from the liver’s Kupffer cells and endothelial cells, we observed significant LAMP1 knockdown in leukocytes in the spleen (*P* < 0.0001) and bone marrow (*P* = 0.0004) as compared with an aNP containing scrambled siRNA (siCtrl), which is mainly attributed to LAMP1 silencing in myeloid cells (Fig. 1n). The in vivo aNP-siLAMP1 silencing potency was on par with an LNP-siLAMP1 formulation used as a positive control (Supplementary Table 1).

## aNP library screening

**Fig. 2:**
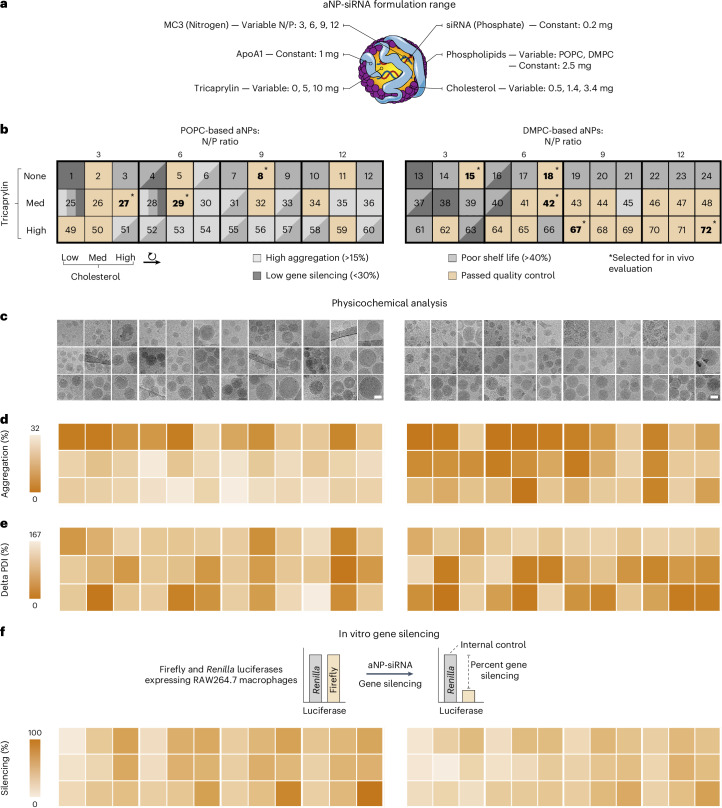
Establishing and screening a library of aNP-siRNA with diverse compositions. **a**, aNP-siRNA library design space. N, nitrogen; P, phosphate. **b**, Compositions, identifiers, and characteristics of the aNP-siRNA formulations in the library. Formulations in bold and indicated by an asterisk (*) were selected for in vivo evaluation. **c**, Morphology and size were determined by cryo-EM analysis (see Supplementary Fig. 2 for extended cryo-EM data). Scale bars, 50 nm. **d**–**f**, Heatmaps represent formulation quality as assessed by percentage lipid aggregation quantified in cryo-EM images (**d**), shelf-life by percentage change in dispersity determined by dynamic light scattering after 28 days (**e**), and functional Fluc reporter gene silencing in vitro determined by bioluminescence measurements in RAW 264.7 dual-luciferase reporter cells after 48 h (**f**). Source data

After prototyping the aNP-siRNA concept, we designed an aNP optimization strategy involving an iterative design process that resulted in a library of 72 compositionally distinct aNP-siRNA formulations (Supplementary Table 1). Within the library, we kept siRNA and apoA1 levels constant while varying the amount of cholesterol, tricaprylin and the MC3 ionizable cationic lipid (Fig. 2a). MC3^3,29,30^ complexes negatively charged siRNA to enable its integration into an aNP’s lipophilic core, whereas cholesterol and tricaprylin provide structural features that are essential for an aNP’s supramolecular organization and structural stability. Amphiphilic phospholipids are required to disperse the lipophilic supramolecular nanoaggregates of siRNA, MC3, tricaprylin and cholesterol in an aqueous environment and, together with apoA1, stabilize the aNP. We selected three phospholipids that are known to properly interact with apoA1^31^, namely, DMPC, 1-palmitoyl-2-oleoyl-glycero-3-phosphocholine (POPC), and 1,2-dipalmitoyl-*sn*-glycero-3-phosphocholine (DPPC). After formulating over two dozen DPPC-based aNPs, we did not identify stable formulations with adequate in vitro silencing features (Supplementary Fig. 1) and decided to exclude these formulations from the library and further testing.

The different aNP compositions and quality features are schematically depicted in Fig. 2b. We observed that increased tricaprylin improved DMPC-based formulations, but not aNPs containing POPC. Cryo-EM (Fig. 2c) revealed that tricaprylin inclusion promoted the formation of spherical structures, but we also found other morphologies such as aNP_39_. In the absence of tricaprylin (aNP_1–24_), cryo-EM revealed an array of different morphologies, ranging from spherical (for example, aNP_4,7,10,11_), to multilamellar (for example, aNP_3,6,15_), to elliptical (for example, aNP_30_); there were also less well defined shapes such as aNP_18_ and aNP_21_ (Supplementary Fig. 2 and Supplementary Table 2).

As a critical quality feature, we measured aNP aggregation directly after production by semi-quantitative assessment of cryo-EM images (Supplementary Fig. 3). We found 55 formulations to pass our predefined threshold of <15%. Using DLS, we also determined the size dispersity and stability of the aNPs when stored at 4 °C for up to 28 days. Interestingly, we observed little correlation between initial aggregation (Fig. 2d) and shelf-life stability (Fig. 2e). Finally, we measured the gene silencing capacity of all 72 aNP formulations in vitro (Fig. 2f). We observed diverse gene knockdown capabilities at a fixed concentration of 100 nM siRNA, ranging from no silencing to nearly complete gene silencing. As nanoparticles’ silencing capacity in vitro is a poor predictor of in vivo silencing following intravenous administration^32^, we primarily used this silencing metric as a quality control for aNP-siRNA functionality.

By setting the quality control limits to 15% for aggregation, a maximum increase of 40% in the polydispersity index (PDI) at 28 days, and at least 30% silencing in vitro, we identified 30 aNPs with potentially favourable in vivo features. From those 30 formulations, we selected eight functional aNPs representative of the library’s diversity (Fig. 2b, in bold) to be included in the ensuing in vivo gene silencing experiments in mice. We included three POPC-based formulations—one of which did not contain tricaprylin—and five DMPC-based formulations with varying quantities of tricaprylin. The morphologies, compositions and nitrogen-to-phosphate ratios of the formulations vary throughout this selection of eight aNPs (Fig. 2 and Supplementary Table 1).

## Functionally screening aNPs in vivo

**Fig. 3:**
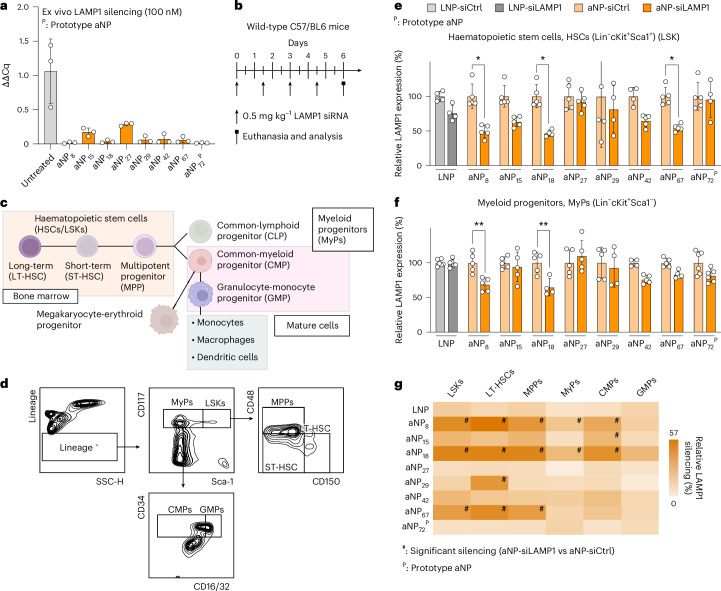
Screening aNP-siRNA functional gene silencing in vivo*.* **a**, Ex vivo LAMP1 silencing in BMDMs via transfection with aNP-siLAMP1. The normalized, relative gene expression levels of *Lamp1* RNA (ΔΔCq) were determined by RT-qPCR. The primers are listed in Supplementary Table 3. Data represent mean ± s.d. of one experiment (*n* = 3 donors). **b**, Schematic screening workflow involving mice receiving repeated intravenous administrations of aNP containing siCtrl or siLAMP1 (0.5 mg kg^–1^ per administration, cumulative dose of 2 mg kg^–1^). LAMP1 expression was determined six days after the first administration by flow cytometry^18^. **c**, Schematic overview of haematopoietic stem cells, myeloid progenitor cells and mature myeloid cells. **d**, Flow cytometry gating strategy (refer to Supplementary Fig. 4 for an extended overview of the gating strategy). Antibodies used for flow cytometry studies are listed in Supplementary Tables 5–8. **e**,**f**, Relative LAMP1 levels indicated by normalized expression levels (%) in haematopoietic stem cells (**e**) and myeloid progenitors (**f**) in the bone marrow. Data represent mean ± s.d. of one experiment (*n* = 4–6 mice) and analysed by one-way ANOVA with a Bonferroni post-hoc test. Statistically significant differences between aNP-siLAMP1 versus aNP-siCtrl formulations are indicated by: *P ≤ 0.05, **P ≤ 0.01, ***P ≤ 0.001. **g**, Heatmap representing relative LAMP1 silencing of selected aNP-siLAMP1 versus aNP-siCtrl formulations in haematopoietic stem and progenitor cells. Colour indicates the measure of silencing and statistically significant differences (*P* ≤ 0.05) are indicated by a hash (#). The data were analysed by one-way ANOVA with a Bonferroni’s post-hoc test. Source data

**Extended Data Fig. 4:**
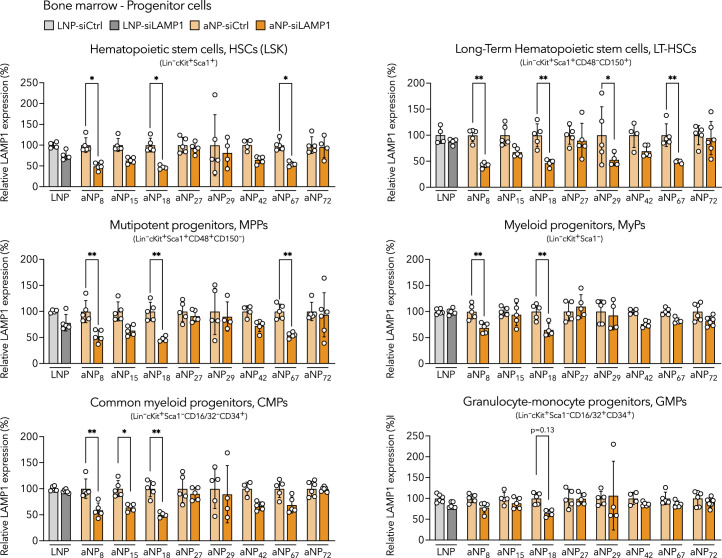
*In vivo* aNP-induced LAMP1 knockdown effect in bone marrow progenitor cells. Relative LAMP1 expression levels measured by flow cytometry in various cell populations in the bone marrow including HSCs, LT-HSCs, MPPs, MyPs, CMPs & GMPs. The gating strategy utilized in this experiment is displayed in Supplementary Fig. 4a. The knockdown effect compared to a negative control formulation is displayed. Data represent mean ± SD of one experiment (n = 6 animals) and analyzed by one-way ANOVA with Bonferroni post-hoc test. Statistically significant differences between aNP-siLAMP1 versus aNP-siCtrl formulations are indicated by: * indicates statistical significance (p ≤ 0.05), ** indicates statistical significance (p ≤ 0.01). Source data

**Extended Data Fig. 5:**
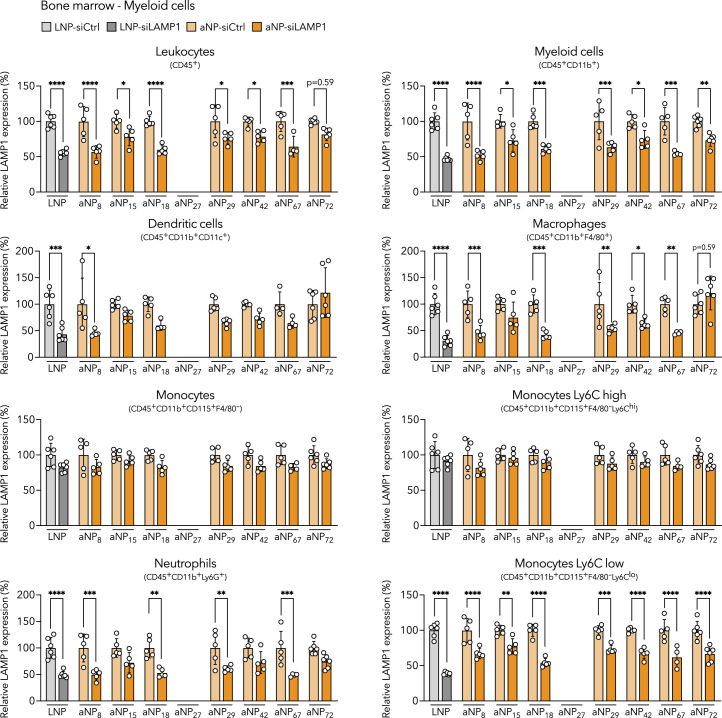
*In vivo* aNP-induced LAMP1 knockdown effect in bone marrow myeloid cells. Relative LAMP1 expression levels measured by flow cytometry in various cell populations in the bone marrow among which leukocytes, myeloid cells, dendritic cells, macrophages, monocytes, monocytes Ly6C high, neutrophils & monocytes Ly6C low. The gating strategy is displayed in Supplementary Fig. 4b. Data represent mean ± SD of one experiment (n = 6 animals) and analyzed by one-way ANOVA with Bonferroni post-hoc test. Statistically significant differences between aNP-siLAMP1 versus aNP-siCtrl formulations are indicated by: * indicates statistical significance (p ≤ 0.05), ** indicates statistical significance (p ≤ 0.01), *** indicates statistical significance (p ≤ 0.001), **** indicates statistical significance (p ≤ 0.0001). Source data

**Extended Data Fig. 6:**
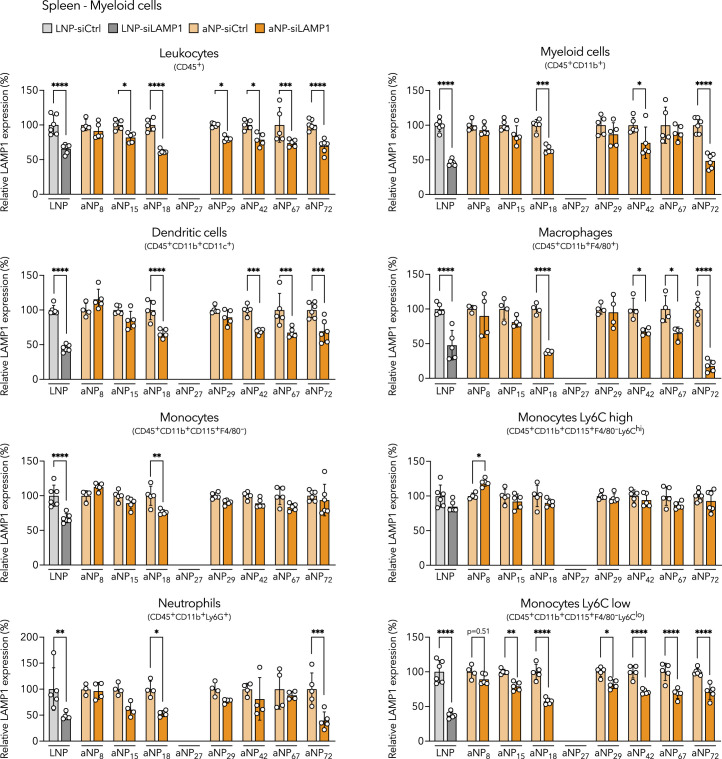
*In vivo* aNP-induced LAMP1 knockdown effect in splenic myeloid cells. Relative LAMP1 expression levels measured by flow cytometry in various cell populations in the bone marrow among which leukocytes, myeloid cells, dendritic cells, macrophages, monocytes, monocytes Ly6C high, neutrophils & monocytes Ly6C low. The gating strategy is displayed in Supplementary Fig. 4b. Data represent mean ± SD of one experiment (n = 6 animals) and analyzed by one-way ANOVA with Bonferroni post-hoc test. Statistically significant differences between aNP-siLAMP1 versus aNP-siCtrl formulations are indicated by: * indicates statistical significance (p ≤ 0.05), ** indicates statistical significance (p ≤ 0.01), *** indicates statistical significance (p ≤ 0.001), **** indicates statistical significance (p ≤ 0.0001). Source data

Following aNP library characterization and in vitro screening, we selected eight representative aNPs for in vivo testing in mice, including the aNP prototype (aNP_72_). Similar to the experiments we conducted with the aNP prototype, we encapsulated siLAMP1 in the selected aNPs. We first assessed the knockdown functionality of these eight aNP-siLAMP1 formulations using bone-marrow-derived macrophages (BMDM) from mice ex vivo. At 100 nM siLAMP1, reverse transcription-quantitative polymerase chain reaction (RT-qPCR) measurements disclosed that all eight aNPs substantially reduced LAMP1 expression compared with the untreated cells (Fig. 3a and Extended Data Fig. 3b). After establishing their functionality, we intravenously administered the eight different aNP-siLAMP1 formulations to mice four times at 36 h intervals, with a siLAMP1 dose of 0.5 mg kg^–1^ (Fig. 3b). Following this aNP-siLAMP1 treatment regimen, we sacrificed the mice and collected bone marrow cells. These cells were stained with flow cytometry antibody panels that we optimized for the detection of haematopoietic stem cells, myeloid progenitors and mature myeloid cells (Fig. 3c,d and Supplementary Fig. 4). Within the aNP selection, we found substantial variability in LAMP1 silencing features in immune cells (Fig. 3e,f and Extended Data Figs. 4–6). Although a clinically approved MC3-based LNP formulation did not induce significant (*P* > 0.9999) LAMP1 knockdown in HSPCs, we observed significant LAMP1 silencing when using aNP_8_ (*P* = 0.0107), aNP_18_ (*P* = 0.0117) and aNP_67_ (*P* = 0.0306). Although aNP_8_ (*P* = 0.0084) and aNP_18_ (*P* = 0.0038) also significantly silenced LAMP1 in myeloid progenitor cells, we did not observe significant LAMP1 knockdown in this progenitor subset using aNP_67_ (*P* = 0.5506). Refer to Fig. 3g for a comprehensive overview of the in vivo silencing features of the different aNP-siLAMP1 formulations. On the basis of these data, aNP_8_ and aNP_18_ formulations showed potential for broad gene silencing of stem and progenitor populations. At the same time, aNP_67_ displays a silencing bias towards stem cells. In light of aNP_18_’s broad silencing features, we selected this formulation for further studies.

## aNP-siRNA profiling and application

**Fig. 4:**
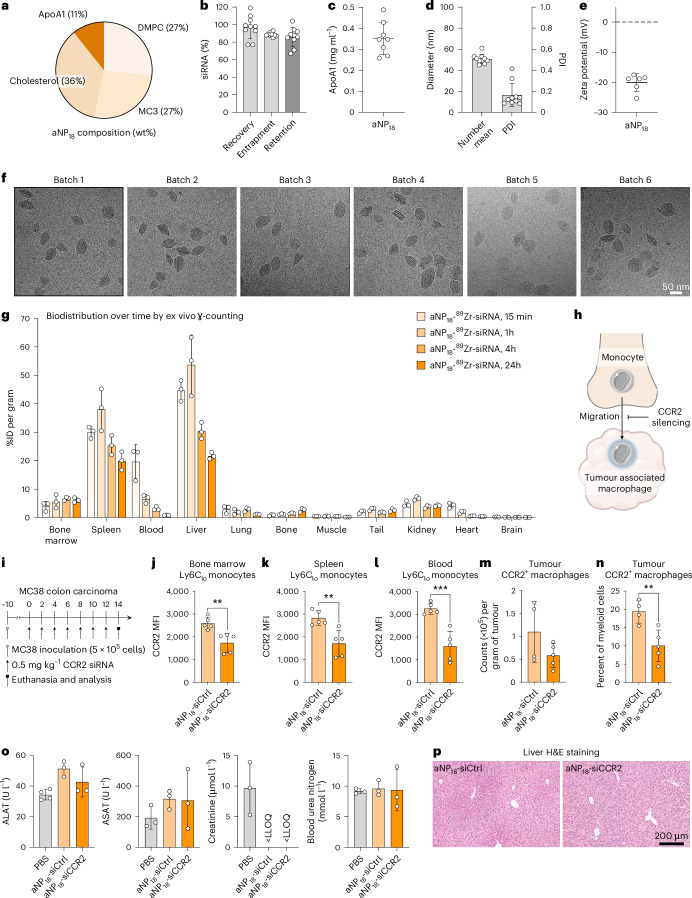
In-depth analysis of lead siRNA-aNP physicochemical properties, in vivo behaviour and therapeutic application. **a**, Lead aNP-siRNA (aNP_18_) composition (wt%). **b**, siRNA recovery, entrapment and retention, data represent mean ± s.d. of one experiment (*n* = 12 formulation batches). **c**, Apolipoprotein A1 (apoA1) content. Data represent mean ± s.d. of one experiment (*n* = 9 formulation batches). **d**, Hydrodynamic diameter represented as the number mean and dispersity. Data represent mean ± s.d. of one experiment (*n* = 12 formulation batches). **e**, Zeta potential. Data represent mean ± s.d. of one experiment (*n* = 6 formulation batches). **f**, Cryo-EM analysis of six individual aNP_18_-siRNA batches. **g**, Quantitative biodistribution of aNP_18_-^89^Zr-siRNA 15 min, 1 h, 4 h and 24 h after intravenous administration in mice (8 µCi per mouse) determined by ex vivo gamma counting. Radioactivity is expressed as %ID per gram. Data represent mean ± s.d. of one experiment, and each data point represents one animal (*n* = 3 mice). **h**, Schematic showing therapeutic effect of CCR2 silencing by inhibiting immunosuppressive monocyte migration to the tumour microenvironment. **i**, Schematic treatment regimen involving MC38-tumour-bearing mice (*n* = 5 mice per group) receiving repeated intravenous administrations of aNP_18_ containing scrambled siRNA (aNP_18_-siCtrl) or CCR2 siRNA (aNP_18_-siCCR2) (0.5 mg kg^–1^ per administration, cumulative dose of 3.5 mg kg^–1^). CCR2 expression was determined 14 days after the first administration by flow cytometry. **j**–**l**, CCR2 levels indicated by mean fluorescence intensity (MFI) in ly6C_lo_ monocytes in the bone marrow (**j**), spleen (**k**) and blood (**l**). Data represent mean ± s.d. of one experiment (*n* = 5 mice) and were analysed using Student's *t*-test. Statistically significant differences between aNP_18_-siCCR2 versus aNP_18_-siCtrl formulations are indicated by: **P ≤ 0.01, ***P ≤ 0.001. **m**, Number of CCR2^+^ macrophages per gram of tumour tissue as determined by flow cytometry. Data represent mean ± s.d. of one experiment (*n* = 5 mice) **n**, Fraction of CCR2^+^ macrophages within tumour tissue expressed as percentage (%) of myeloid cells. Data represent mean ± s.d. of one experiment (*n* = 5 mice) and analysed using Student's *t*-test. Statistically significant differences between aNP_18_-siCCR2 versus aNP_18_-siCtrl formulations are indicated by: **P ≤ 0.01. **o**, Biocompatibility of siRNA-aNP_18_-siCCR2 as determined by serum levels of alanine aminotransferase (ALAT, left), aspartate aminotransferase (ASAT, middle left), creatinine (middle right) and urea (right) two days following treatment (cumulative dose of 3.5 mg kg^–1^). Data represent mean ± s.d. of one experiment (*n* = 3 or 4 mice). LLOQ, lower limit of quantification. **p**, Representative liver sections stained with haematoxylin and eosin from mice treated with aNP_18_-siCtrl (left) and aNP_18_-siCCR2 (right). Source data

After identifying broad gene silencing features (Figs. 2 and 3), we selected aNP_18_ for in-depth analysis. aNP_18_ is a DMPC-based formulation that does not contain tricaprylin (Fig. 4a). It demonstrates siRNA recovery, entrapment and retention values of over 80% (Fig. 4b) while maintaining consistent apoA1 levels (Fig. 4c) across multiple batches. aNP_18_ has a hydrodynamic diameter of approximately 50 nm, with a narrow size distribution (PDI < 0.2; Fig. 4d), and zeta potential of −20 mV (Fig. 4e). Notable, its siRNA entrapment, hydrodynamic diameter and dispersity remained stable for eight weeks when stored in phosphate-buffered saline (PBS) at 4 °C (Extended Data Fig. 9a). Furthermore, cryo-EM data revealed that aNP_18_-siRNA predominantly consists of multilamellar, elliptical structures, which was consistent across six independently produced batches (Fig. 4f).

**Extended Data Fig. 7:**
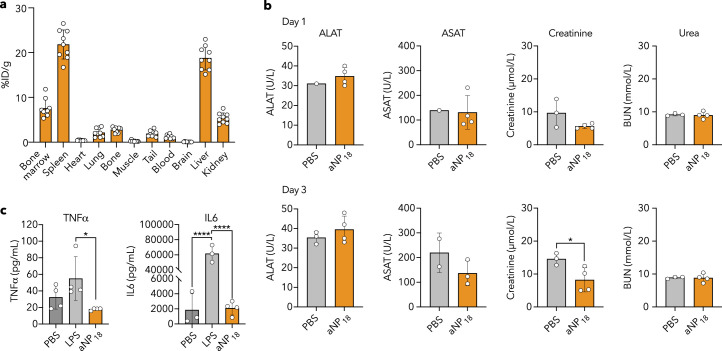
aNP biodistribution and tolerability following single i.v. injection. **a**. Quantitative biodistribution of aNP_18_-^89^Zr-siRNA 24 h following intravenous administration in mice (8 µCi/mouse) determined by gamma counting. Radioactivity is expressed as the percentage injected dose per gram of tissue (%ID/g). Data represent mean ± SD of one experiment, each data point represents one animal (n = 9 mice). **b**. Biocompatibility of siRNA-aNP_18_ as determined by serum levels of alanine aminotransferase (ALAT), aspartate aminotransferase (ASAT), creatinine, and urea one day (*top*) and 3 days (*bottom*) after intravenous administration (0.5 mg/kg) (n = 1-4 mice). Data represent mean ± SD of one experiment (n = 1-4 mice). **c**. Cytokine levels were determined by ELISA 4 h after intravenous administration of siRNA-aNP_18_ and intraperitoneal administration of LPS (0.5 mg/kg). Data represent mean ± SD of one experiment (n = 3-4 mice) and analyzed by one-way ANOVA with Bonferroni post-hoc test. Source data

Using the same method that we applied for the prototype aNP_72_ (Fig. 1i), we integrated ^89^Zr-siRNA into aNP_18_ to assess its biodistribution in mice at various time points following intravenous administration using ex vivo gamma counting (Fig. 4g). For aNP_18_, we observed considerable bone marrow accumulation and approximately twofold-higher uptake in the spleen compared with LNPs after 24 h (Fig. 1m and Extended Data Fig. 7a). As the spleen also contains myeloid and progenitor cells, the high uptake in this organ is beneficial. The liver uptake—which we found to be slightly lower than for LNPs 24 h after administration (Fig. 1m)—and kidney accumulation must be considered and carefully monitored. We therefore measured liver enzymes and kidney toxicity markers, and did not observe substantial differences after PBS or aNP_18_-siRNA injections (Extended Data Fig. 7b). Furthermore, a single aNP_18_-siRNA administration resulted in tumour necrosis factor alpha (TNFα) and interleukin-6 (IL-6) concentrations comparable with mice that received intravenous PBS, and significantly lower concentrations (*P* = 0.0432 for TNFα and *P* < 0.0001 for IL-6) than mice receiving intraperitoneal LPS, used as positive control (Extended Data Fig. 7c).

**Extended Data Fig. 8:**
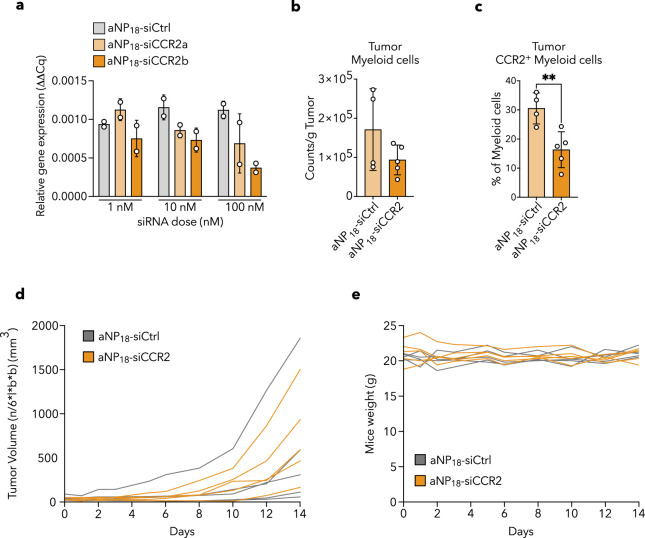
*Ex vivo* CCR2 silencing and *in vivo* biological effects in aNP-treated MC38 tumour-bearing mice. **a**. Screening of siCCR2 constructs (Supplementary Table 4) using bone marrow-derived macrophages. Transfection was performed with aNP_18_-siCCR2 or aNP_18_-siCtrl, at concentrations 1, 10, and 100 nM siRNA, respectively. Expression levels of *Ccr2* were determined by RT-qPCR and the ΔΔCq-values are displayed. The primers are listed in Supplementary Table 3. Data represents mean ± SD of one experiment (minimal of n = 2 biological replicates). **b**. Number of myeloid cells per gram of tumour tissue as determined by flow cytometry. Data represent mean ± SD of one biological replicate (n = 4-5 mice) and analyzed by student’s t-test **c**. Fraction of CCR2-positive myeloid cells within tumour tissue expressed as percentage (%) of myeloid cells. Data represent mean ± SD of one experiment (n = 4-5 mice) and analyzed by student’s t-test. Statistically significant differences between aNP_18_-siCtrl versus aNP_18_-siCCR2 formulations are indicated by: ** indicates statistical significance (p = 0.0084). **d**. Individual tumour volumes of MC38 tumour-bearing mice (n = 5 per group) receiving repeated intravenous administrations of aNP_18_ containing scrambled siRNA (aNP_18_-siCtrl) or CCR2 siRNA (aNP_18_-siCCR2) (0.5 mg/kg per administration, cumulative dose 3.5 mg/kg). **e**. Individual mouse body weights (n = 5 mice). Source data

We next evaluated aNP_18_’s therapeutic potential by silencing C–C chemokine receptor type 2 (*Ccr2*) expression in a syngeneic tumour mouse model. LNP-siRNA targeting *Ccr2* mRNA (siCCR2) treatment has previously been shown to decrease influx of myeloid cells in tumours, affecting the immunosuppressive tumour microenvironment^33^ (Fig. 4h). We therefore formulated aNP_18_ with two siCCR2 constructs that induced CCR2 knockdown in murine BMDMs ex vivo compared with aNP-siCtrl (Extended Data Fig. 8a). Finally, using the most potent siCCR2 construct, we administered aNP_18_-siCCR2 intravenously to MC38 tumour-bearing C57/BL6 mice (Fig. 4i). Treatment with aNP_18_-siCCR2 induced significant CCR2 knockdown in ly6C_lo_ monocytes of the bone marrow (*P* = 0.005), spleen (*P* = 0.0056) and blood (*P* = 0.0007) (Fig. 4j–l). Simultaneously, and in line with previous findings^33^, we observed a considerable decrease of tumour-resident CCR2^+^ myeloid cells (Extended Data Fig. 8b,c) and macrophages following aNP_18_-siCCR2 treatment compared with aNP_18_-siCtrl (Fig. 4m,n), without impacting tumour size (Extended Data Fig. 8d). As these myeloid cells are generally immunosuppressive^34^, their reduced presence indicates a decreased influx of monocytes from the haematopoietic organs, indicative of the aNP platform’s potential for immunotherapy. Furthermore, we did not observe notable changes in body weight (Extended Data Fig. 8e), liver enzyme levels, kidney toxicity markers (Fig. 4o), or damage using histological analyses of liver sections, following treatment with aNP_18_-siCtrl and aNP_18_-siCCR2 (Fig. 4p and Supplementary Fig. 5).

## aNPs functionally deliver ASO and mRNA

**Fig. 5:**
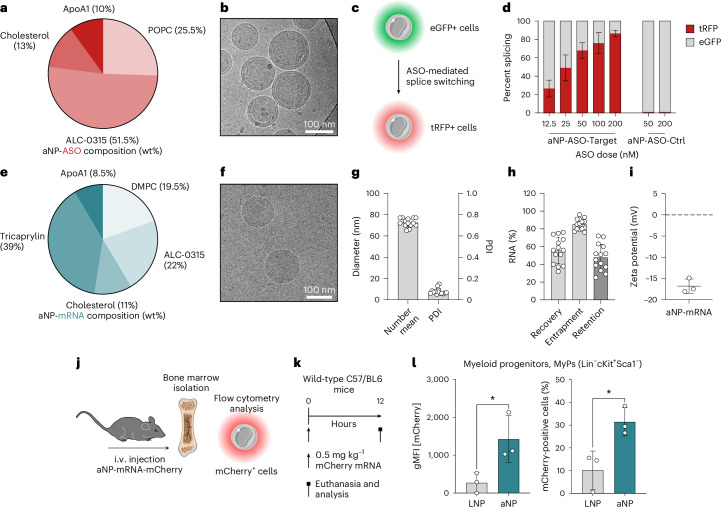
aNP formulations for ASO and messenger RNA delivery to immune cells. **a**, aNP-ASO composition (wt%). **b**, Cryo-EM image. **c**, Schematic of NATURA reporter cells that alter the expression of eGFP to tRFP upon ASO-mediated splice-switching^37^. **d**, Splice-switching in RAW 264.7 reporter cells in vitro. Data represent mean ± s.d. of two experiments with two formulation batches (*n* = 2). **e**, aNP-mRNA composition (wt%). **f**, Cryo-EM image. **g**, Hydrodynamic diameter represented as the number mean and dispersity. Data represent mean ± s.d. of one experiment with 14 formulation batches (*n* = 14). **h**, mRNA recovery, entrapment and retention data represent mean ± s.d. of one experiment (*n* = 14 formulation batches). **i**, Zeta potential data represent mean ± s.d. of one experiment with three individual formulations (*n* = 3 formulations). **j**,**k**, Schematic workflow of intravenously (i.v.) administering aNPs containing mCherry mRNA (aNP-mRNA-mCherry) (**j**) at a dose of 0.5 mg kg^–1^ mRNA to C57/BL6 mice and determining expression in bone marrow cells by flow cytometry analysis (**k**). **l**, mCherry expression in bone marrow myeloid progenitors indicated by geometric mean fluorescence intensity (gMFI, left panel) and percentage of mCherry^+^ cells (right panel) 12 h after intravenous administration with LNP and aNP containing mCherry mRNA. Data represent mean ± s.d. of one experiment (*n* = 3 mice) and analysed by student t-test. Statistically significant differences between LNP-mRNA versus aNP-mRNA formulations are indicated by: *P ≤ 0.1. Source data

Aside from gene silencing, we explored the aNP platform’s capacity for delivering other types of nucleic acid therapeutics. Using the approach we established for the prototype aNP_72_-siRNA, we first conducted pilot experiments to identify stable aNP formulations containing the ionizable cationic lipid ALC-0315 (refs. ^4,35^). Using a tailored formulation containing POPC, cholesterol and ALC-0315, we incorporated an ASO that induces splice-switching^36^ at an N/P ratio of 9 (Fig. 5a). The aNP-ASO formulation’s mean hydrodynamic diameter was approximately 120 nm with a narrow size distribution (PDI < 0.1; Extended Data Fig. 10b). Cryo-EM corroborated this finding and uncovered core–shell structures (Fig. 5b). In line with aNP-siRNA formulations, ASO was stably incorporated, with entrapment and recovery values of around 80% (Extended Data Fig. 10c). To determine the aNP-ASO’s functional splice-switching effects in vitro, we transfected RAW 264.7 cells with the NATURA reporter^37^. These cells stably express enhanced green fluorescent protein (eGFP), which changes to turbo red fluorescent protein expression (tRFP) upon successful blocking of a frameshift exon by a specific ASO (Fig. 5c and Extended Data Fig. 10a). Following the reporter cells’ exposure to aNPs containing frameshift-targeted ASO, we observed dose-dependent splice-switching effects (Fig. 5d). We observed no splice-switching for the control aNP.

**Extended Data Fig. 9:**
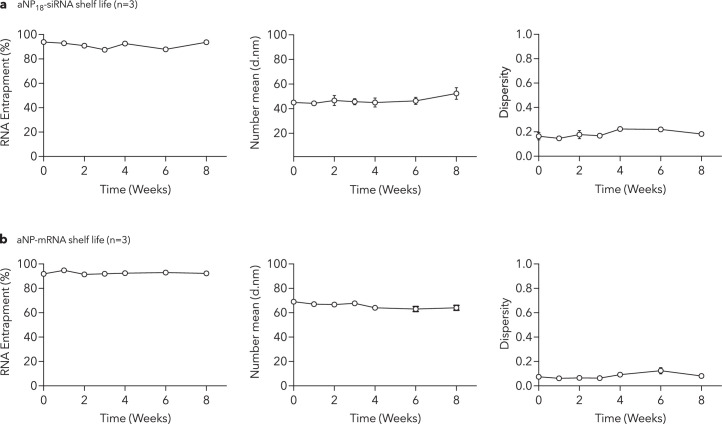
aNP-siRNA and aNP-mRNA shelf-life. **a**. aNP_18_-siLAMP1’s RNA entrapment (left), hydrodynamic diameter presented as the number mean (middle), and associated dispersity (right) over a period of 8 weeks while stored at 4 °C. Data represents mean ± SD of one experiment with 3 formulation batches (n = 3 formulation batches). **b**. aNP-mRNA’s RNA entrapment (left), hydrodynamic diameter presented as the number mean (middle), and associated dispersity (right) over a period of 8 weeks while stored at 4 °C. Data represents mean ± SD of one experiment with 3 formulation batches (n = 3 formulation batches). Source data

**Extended Data Fig. 10:**
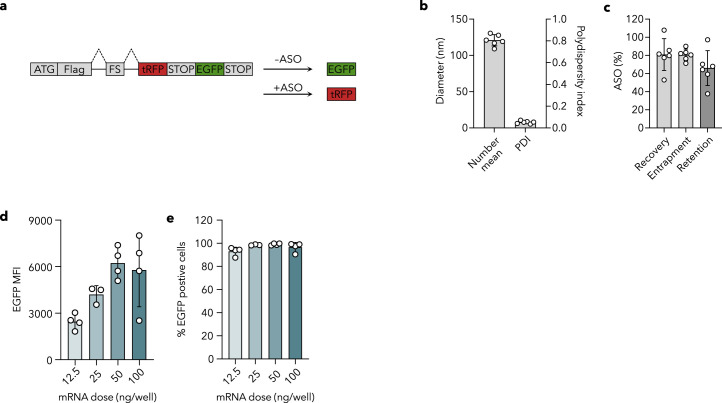
aNP-ASO physicochemical properties and aNP-mRNA *in vitro* eGFP expression. **a**. Schematic of the NATURA reporter system^37^. NATURA reporter cells express eGFP, and switch to tRFP after ASO-induced splice-switching. aNP-ASO’s physicochemical characterization: **b**. Hydrodynamic diameter presented as number mean and associated dispersity, data represent mean ± SD of one experiment (n = 6 formulation batches). **c**. ASO recovery, entrapment and retention, data represent mean ± SD of one experiment (n = 6 formulation batches). In vitro transfection of RAW 264.7 macrophages with aNP-mRNA-encoding eGFP. **d**. eGFP MFI as characterized by flow cytometry. Data represent mean ± SD of 4 experiments with 4 individual batches of aNPs(n = 4). **e**. Percentage (%) eGFP positive cells as characterized by flow cytometry. Data represent mean ± SD of 4 experiments with 4 individual batches of aNPs (n = 4). Source data

To evaluate the aNP platform’s ability to encapsulate and deliver mRNAs, we designed an aNP containing DMPC, cholesterol, triglycerides and ALC-0315 using an N/P ratio of 6 (Fig. 5e). Cryo-EM revealed aNP-mRNA had a spherical morphology and a diameter of about 70 nm (Fig. 5f), which was confirmed by DLS (Fig. 5g). We also found mRNA entrapment to be efficient (>80%) and constant between various aNP-mRNA batches (Fig. 5h). Importantly, the aNP formulation’s mRNA entrapment, hydrodynamic diameter and dispersity remained stable for eight weeks when stored in PBS at 4 °C (Extended Data Fig. 9b). The aNP-mRNA’s zeta potential of approximately −17 mV was comparable with aNP_18_-siRNA (Figs. 5iand 4e). To evaluate mRNA translation in vitro, we treated RAW 264.7 cells with aNPs containing mRNA-encoding GFP. Flow cytometry analysis revealed efficient and dose-dependent transfection efficiency (Extended Data Fig. 10d,e). Finally, we evaluated aNP-mRNA’s functional effects in vivo by intravenously administering aNPs and LNPs containing mRNA-encoding mCherry. Flow cytometry analyses of bone marrow myeloid progenitors (Fig. 5j,k) revealed significantly more mCherry^+^ cells (*P* = 0.0241) and higher expression levels (*P* = 0.0407) following treatment with aNP-mRNA when compared to LNP-mRNA (Fig. 5l).

## Conclusion

Lipid molecules such as cholesterol are transported throughout the body via endogenous nanoparticles known as lipoproteins^20^. For example, HDL can accept cholesterol from myeloid cells such as macrophages in inflammatory tissues—a process mediated by passive diffusion and via ATP-binding cassette transporters and scavenger receptors^38^. Aside from interacting with mature myeloid cells, HDL has been shown to directly interact with HSPCs^39^. Although lipoproteins fulfill many important tasks in lipid metabolism^40,41^, HDL has also been implicated in transporting RNAs in the human body^21^. Owing to these diverse features, HDL and its trafficking in the human body provide a compelling framework for RNA delivery to immune cells and their progenitors in the bone marrow. Although reconstituted HDL or HDL-mimicking nanoparticles have been developed for delivering cholesterol-conjugated siRNA, this strategy results in hydrophilic siRNA’s surface-integration through the lipophilic cholesterol anchor that is incorporated in the phospholipid layer^42^. This approach has been explored for hepatic delivery via scavenger receptor type B1 (SR-B1)^43^ and vaccine purposes^44,45^. Others have used apolipoproteins containing cationic lipoplexes for RNA delivery, inevitably exposing the payload to the outside environment^46^. Here we presented the development and evaluation of the aNP platform that stably incorporates a variety of nucleic acid payloads in its core. By varying the aNP composition (Supplementary Table 1), we can finetune properties to optimize RNA delivery to diverse myeloid cells, as well as HSPCs in the bone marrow. Although LNPs have been shown to reach bone marrow endothelial cells^47–49^, compositional modifications^19,50^ or antibody conjugation strategies^51,52^ are generally required for efficient HSPC targeting. Importantly, it should be considered that LNP technology was not explicitly designed for immune cell targeting. Conversely, we specifically designed the aNP platform with immune cell and HSPC targeting in mind and achieved gene regulation in these cell subsets. On the basis of our previous studies^53^, we assume aNP interactions with myeloid cells and their bone marrow progenitors are primarily mediated via apoA1. However, we cannot exclude aNPs containing a small fraction of other apolipoproteins due to the apoA1 isolation protocol we have used for this study. Besides gene silencing using siRNA, aNPs are suitable for delivering functional ASOs and mRNA. We foresee aNP delivery technology progressing into a designer platform for therapeutic immunoregulation in a wide range of immune-mediated diseases, for diverse vaccination applications, and for gene HSPC editing in certain hereditary diseases. The aNP platform’s natural features ensure high biocompatibility and allow for repeated dosing, which, in conjunction with its modularity and the ability to scale production, make it attractive for clinical translation.

## Methods

### Materials

- POPC (Avanti Polar Lipids, 850457)
- DMPC (Avanti Polar Lipids, 850345)
- DSPC (Avanti Polar Lipids, 850365)
- Cholesterol (Sigma-Aldrich)
- Tricaprylin (Sigma-Aldrich)
- PEG(2K)-DMG (Avanti Polar Lipids, 880151)
- DLin-MC3-DMA (SymoChem)
- Sodium acetate (Sigma-Aldrich)
- 12–14 kDa molecular weight cut-off (MWCO) dialysis membrane, Spectra/Por
- Quant-iT RiboGreen reagent (Thermo Fisher Scientific)
- TE buffer (10 mM Tris-HCl, 1 mM, EDTA, pH 7.5 in DEPC-treated water)
- Apolipoprotein A1 FS assay (DiaSYS)
- Spin filters 100,000 Da MWCO (Amicon)
- LabAssay phospholipids (Fujifilm, Wako)
- Cholesterol FS assay (Diasys)
- Dual-Luciferase Reporter Assay System (Promega)
- HDL plasma fraction (Medix Biochemica)
- RAW 264.7 murine macrophages (ATCC TIB-71)
- Lipofectamine RNAiMAX (Thermo Fisher Scientific)
- Deferoxamine-DBCO (Macrocyclics)
- 2 kDa MWCO Dialysis tubing (Sigma-Aldrich)
- Corning 70 µm cell strainer (Merck)
- CD11b MicroBeads (Miltenyi Biotec)

### aNP production

Apolipoprotein nanoparticles containing nucleic acids were formulated by rapid T-junction mixing as previously described in ref. ^28^. The lipid molecules (POPC; DMPC or DSPC; cholesterol; tricaprylin (PEG-DMG for LNP particles); and DLin-MC3-DMA) were dissolved in ethanol at ratios specified in Supplementary Table 1. siRNA, mRNA or ASO were dissolved in 25 mM sodium acetate (pH 4). Next, organic and aqueous phases were mixed through a T-junction at a flow rate ratio of 1:3 (organic-to-aqueous) and collected in a 12–14 kDa MWCO dialysis membrane (Spectra/Por). Nanoparticle formulations were dialysed against 1× PBS (pH 7.4) for 4 h and refreshed for dialysis overnight at 4 °C while stirred at 150 r.p.m. Excess organic and aqueous phases were stored at 4 °C for physicochemical analysis. On the next day, samples were collected from the dialysis bags and the volume was determined. For the aNP particles, the appropriate amount of apolipoprotein A1 (apoA1) in PBS was diluted to one-third of the nanoparticle sample volume. ApoA1 was introduced to the nanoparticle formulations by rapid T-junction mixing at a flow rate ratio of 1:3 (apoA1 solution/nanoparticle solution). After apoA1 addition, samples were incubated at room temperature for 1 h for aNP-siRNA and aNP-ASO, and 20 min for aNP-mRNA. The resulting product was filtered through a 0.2 μm filter, and concentrated and purified by centrifugal filtration using a 100 kDa MWCO filter. Samples were diluted to the desired siRNA, ASO or mRNA concentration and stored at 4 °C until further use. Aseptic techniques were used after 0.2 μm filtration.

### aNP physicochemical analysis

The nanoparticle hydrodynamic diameter (expressed as the number-weighted mean diameter) and zeta potential were determined by DLS using a Zetasizer Nano ZS (Malvern Instruments) equipped with a Zetasizer NanoSampler (Malvern Instruments). Size dispersity was measured as the PDI. For DLS measurements, 100 µl of the formulation sample was diluted into 700 µl of 1× PBS and equilibrated at room temperature before analysis. Each sample was measured five times (10 runs of 10 s) without fixing the attenuator and measurement position. For zeta potential measurements, the sample was diluted 50 times in MilliQ water, and 700 µl of diluted sample was loaded in a disposable folded capillary cuvette (DTS1070, Malvern Panalytical). Five measurements were performed at intervals of 300 s, and each measurement comprised 40 runs with 40 V applied. The Quant-iT RiboGreen assay (Thermo Fisher Scientific) was used to quantify the amount of RNA loaded inside of the formulated particles. A standard formulation sample with a theoretical RNA concentration of 133.3 µg ml^–1^, was diluted 200 times in TE buffer both with and without 2% Triton X-100 in a black 96-well plate to a total volume of 100 µl. The Triton detergent disrupts the lipid-based nanoparticles; therefore, total RNA (retained and unretained) becomes accessible for the Quant-iT RiboGreen reagent. Known RNA controls, stored during formulation, were diluted 53.3 times in TE buffer both with and without 2% Triton to a total volume of 100 µl. Next, RiboGreen reagent was 200-fold diluted using TE buffer both with and without Triton X-100; 100 µl of this dilution was added to each well containing sample or control to bring the total volume to 200 µl. The fluorescence of the samples was then measured on a Tecan Spark microplate reader at an excitation wavelength of 480 nm and emission wavelength of 520 nm. RNA recovery was determined as: (amount of RNA (retained + unretained))/(total amount of RNA (used in the formulation)) × 100%. The entrapment of RNA was defined as: (1-((amount of RNA (retained + unretained))/(total amount of RNA (used in the formulation)))) × 100%. RNA retention was determined as: recovery × entrapment. Cholesterol was quantified using the cholesterol FS assays (DiaSYS). Briefly, 240 µl of buffer containing colour reagent was added to 10 µl of sample in a transparent 96-well plate and incubated at 37 °C for 5 min. Absorbance was measured at 500 nm using a Tecan Spark plate reader. Phospholipids were quantified using the LabAssay Phospholipid (FUJIFILM). For this assay, 190 µl of buffer containing colour reagent is added to 10 µl of the sample in a transparent 96-well plate and incubated at 37 °C for 30 min. The absorbance was measured at 600 nm using a Tecan Spark plate reader. The amount of apoA1 was quantified using the Apolipoprotein A1 FS assay (DiaSYS). In short, 200 µl of Reagent 1 was added to 5 µl sample or control in a transparent 96-well plate. After incubating the plate at 37 °C for 5 min, the absorbance was measured using a Tecan Spark microplate reader at 580 nm. Next, 50 µl of Reagent 2 containing an anti-apoA1 antibody was added and the solution was incubated at 37 °C for 5 min before measuring the absorbance at the same setting.

### ApoA1 isolation from human plasma

Human HDL plasma fraction was purchased from Medix Biochemica (Maryland Heights). Using KBr salt, the density was adjusted to 1.22–1.24 g ml^–1^, and the solution was centrifuged for 48 h, 250,000 × *g* at 4 °C. The gel-like pellets were carefully collected and dissolved overnight in Milli-Q on a roller at 400 r.p.m. and 4 °C to a concentration of 37.5 mg ml^–1^. Next, the HDL solution was dripped at a 0.4 ml min^–1^ into methanol/chloroform (50/50 vol%) on dry ice to separate the lipids from the protein. About 50 mg of HDL precipitated in 240 ml solvent. The solvent containing the protein precipitate was decanted and centrifuged at 300 × *g* for 10 min at 4 °C. The supernatant was carefully discarded, and the whitish apoA1 pellets collected. The pellets were dried in the vacuum oven at 37 °C for 2–3 h to remove residual solvent. The resulting white-yellowish flakes were redissolved in 6 M guanidine solution (5 ml per 50 mg of protein). Finally, isolated protein was dialysed against PBS at 4 °C for one week. Phosphate-buffered saline was refreshed daily (dialysis ratio = 1:100) and 3,500 Da MWCO SnakeSkin dialysis membranes (Thermo Fisher Scientific). The dialysate was collected and quality was assessed via SDS-page, Nanodrop and Apolipoprotein FS assay. ApoA1 was aliquoted, snap-frozen, and stored at −80 °C.

### In vitro gene silencing

In vitro silencing experiments were performed in a RAW 264.7 (ATCC TIB-71) cell line transfected with the pmirGLO plasmid (containing *Renilla* and firefly luciferases expressing gene sequences). Cells were cultured until 80% confluency, detached with trypsin, counted and seeded at 25,000 cells per well in a 96-well plate. After overnight recovery, the cells were transfected with nanoparticles containing either scrambled siRNA (Integrated DNA Technologies, IDT) or anti-firefly luciferase siRNA at the desired concentration. After incubating for 48 h, the medium was washed off with 1× PBS and lysate phosphate buffer (Dual-Luciferase Reporter Assay System, Promega) was added to lyse the cells; 10 µl of the cell lysate was transferred to a white 96-well flat-bottomed plate. Subsequently, 40 µl of ONE-Glo reagent (Dual-Luciferase Reporter Assay System, Promega) was added and luminescence was measured with a Tecan Spark microplate reader at an integration time of 500 ms and settle time of 1,000 ms. A luminescence scan was performed to validate the intensity peak at 560 nm for firefly luciferase; 40 µl of Stop and Glo reagent (Dual-Luciferase Reporter Assay System, Promega) was then added to each well and the luminescence was measured again in the same way. The firefly luminescence was normalized to the *Renilla* luminescence and subsequently expressed as a percentage of the untreated sample signal.

### In vitro exon skipping

Exon skipping experiments were performed on NATURA reporter cells^37^. Upon ASO-induced exon skipping, the reporter cells switch expression from eGFP to tRFP thereby giving quantitative information about the degree of skipping. In short, RAW 264.7_PC2 cells (ATCC TIB-71) were seeded in a 96-well plate at a density of 30,000 cells per well. Cells were left to attach overnight. Next, the cells were transfected with aNPs containing either target or scrambled ASOs at concentrations ranging from 12.5–200 nM ASO; 48 h after transfection, cells were harvested, washed, stained with DRAQ7 (Thermo Fisher Scientific) and resuspended in FACS buffer (1x PBS + 0.5% BSA + 2 mM EDTA). All data were acquired using the BD FACSAria III Cell Sorter (BD biosciences), eGFP (FITC), tRFP (PE) and DRAQ7 (APC). As a positive control, RAW 264.7_PCT cells that continuously express tRFP^37^ were utilized. The protein percentage spliced is calculated according to the following formula: pPSI = (0.558 × MFI tRFP)/(MFI eGFP + (0.558 × MFI tRFP)). The relative brightness of tRFP in relation to eGFP is adjusted by multiplying by a factor of 0.558.

### In vitro mRNA expression

RAW 264.7 (ATCC TIB-71) cells were seeded in a 96-well plate at a density of 30,000 cells per well. The cells were left to attach overnight. Cells were then transfected with aNPs containing eGFP-mRNA (RiboPro) at a concentration range of 0–100 ng per well. Cells were harvested 24 h later and prepared for flow cytometry by washing and staining with DRAQ7 (Thermo Fisher Scientific). Finally, cells were resuspended in FACS buffer (1x PBS + 0.5% BSA + 2 mM EDTA). All data were acquired using the BD FACSAriaTM III Cell Sorter (BD biosciences), eGFP (FITC) and DRAQ7 (APC).

### Cryogenic transmission electron microscopy

Just before the vitrification, the surface of 200-mesh lacey carbon supported copper grids (Electron Microscopy Sciences) was exposed to plasma for 40 s using a Cressington 208 carbon coater. Subsequently, 3 µl of aNP sample was applied on a grid and vitrified into a thin film by plunge vitrification in liquid ethane using an automated robot (FEI Vitrobot Mark IV). Cryo-EM imaging was performed on the cryoTITAN (Thermo Fisher Scientific), equipped with a field emission gun, a post column Gatan imaging filter (model 2002), and a post-GIF 2k × 2k Gatan CCD camera (model 794). The micrographs were acquired at either 6,500× (electron dose of 1.64 electrons Å^−2^ s^−1^) or 24,000× magnification (electron dose of 11.8 Å^−2^ s^−1^) at 300 kV acceleration voltage in the bright-field TEM mode with zero-loss energy filtering and a 1 s acquisition time. The size was quantified by measuring the nanoparticle diameter using ImageJ (NIH). At least 150 particles were analysed for each formulation.

### ApoA1-immunogold labelling of apoA1 on siRNA-aNPs

Thirty minutes before staining, a copper grid (carbon support film, 200 mesh, Electron Microscopy Sciences) was plasma-discharged and placed on a 20 µl droplet of the aNP sample (dilution 1:20) for 3 s with the carbon film facing down. The grid was washed five times by incubating it on a 20 µl droplet of PBS for 2 min each time. Next, it was placed on 20 µl of 1% BSA in PBS for 3 min and then on 20 µl of mouse anti-human monoclonal apoA1 antibody in 1% BSA (dilution 1:200, clone: B10, Santa Cruz Biotechnology) for 45 min. After washing, the grid was incubated on 20 µl of the bridging antibody in 1% BSA (1:200 dilution, polyclonal rabbit anti-mouse IgG, Jackson Immunoresearch) for 20 min, washed and placed on 20 µl of 10 nm protein-A-coated gold nanoparticles (1:25 dilution, Cell Microscopy Core, Department of Cell Biology, Utrecht University Medical Center) for 20 min. Finally, the grids were washed six times in PBS and ten times in demi water (each wash was 20 µl and a 3 min incubation). The grid was then placed on a 20 µl droplet of 2% uranyl acetate in water for 5 min. After these steps, the TEM grids were air-dried overnight. The gold-labelled nanoparticles were visualized at room temperature by electron microscopy. The acquisition parameters were the same as for cryo-EM described above.

### Cryo-EM analysis of lipid aggregation

Lipid aggregates were defined on cryo-EM images at 6,500-times magnification as either spherical lipid structures, larger than the main population nanoparticles and displaying different electron densities, or clusters of nanoparticles (Supplementary Fig. 3). The aggregate area was manually delineated by using a bare-hand drawing tool in ImageJ. The lipid aggregation was expressed as the percentage of aggregates in the total image area. At least ten images were analysed for each formulation.

### Radiolabelling of aNP-siRNA

The siRNA-azide had the following sequence:

5′-rArCrCrCrUrGrArArGrUrUrCrArUrCrUrGrCrArCrCrArCCG/3AzideN/-3′.

5′-rCrGrGrUrGrGrUrGrGrArGrArUrGrArArCrUrUrCrArGrGrGrUrCrA-3′.

siRNA-azide and DFO-DBCO were separately dissolved in Milli-Q water containing 5 vol% DMSO to reach concentrations of 0.5 mg ml^–1^. The siRNA-azide solution (270 μl, 8 nmol) was mixed with the DFO-DBCO solution (129 μl, 7.6 nmol) and incubated in the dark and at room temperature for 16 h. The solution was dialysed (2 kDa MWCO dialysis tube, Sigma-Aldrich) against PBS (1 l). The product was freeze-dried, and product formulation was confirmed by MALDI-TOF-MS. The siRNA-DFO remained stable for at least a year when stored at −80 °C. Next, a ^89^Zr oxalate solution in 1 M oxalic acid was neutralized using a 1 M sodium carbonate solution until a pH between 6.8–7.4 was reached (total volume < 25 μl). The neutralized ^89^Zr solution was added to DFO-siRNA dissolved in Milli-Q water (0.5 ml) and the mixture was incubated at 37 °C using a thermomixer (300 r.p.m.) for 60 min. Completion of the reaction was confirmed by radio-TLC (Typhoon 7000IP plate reader, GE Healthcare). The ^89^Zr-siRNA was directly used to formulate the aNP-^89^Zr-siRNA, which resulted in radiochemical yields of >80%, assessed by radio-TLC. Next, the aNP-^89^Zr-siRNA were analysed by Cryo-EM and DLS, to assess their morphology, size and dispersity, respectively.

### Biodistribution over time

Female C57BL/6 mice were intravenously administered with aNP-^89^Zr-siRNA (8 µCi ^89^Zr correlating to 1–1.5 mg kg^–1^ siRNA) in 150–200 µl PBS via tail-vein injection. At various time points after injection (15 m, 1 h, 4 h and after 24 h), mice were euthanized, perfused with PBS, and tissues (bone marrow, spleen, heart, lung, muscle, tail, blood, brain, liver and kidneys) were collected and weighed. Next, the emitted *ɣ* radiation was measured by a gamma counter (2480 WIZARD2 Automatic Gamma Counter, PerkinElmer). Radioactivity values were corrected for decay and normalized to tissue weight to express radioactivity concentration as %ID per gram.

### PET–CT imaging acquisition and analysis

Positron emission tomography imaging was performed 24 h after aNP-^89^Zr-siRNA injection using an IRIS PET–CT (Inviscan). Mice were anesthetized with a gas mixture of 2% isoflurane and 5% oxygen. A 10 min static whole-body PET scan was conducted using an energy window between 250–750 keV followed by a 20 s whole-body CT scan (energy 80 kV, 0.9 mA, 576 projections, voxel size 160 µm). Reconstruction of the PET images was achieved with the 3D Ordered Subsets Expectation Maximization (3D-OSEM-MC) algorithm (eight subsets and eight iterations) using decay, random and dead-time correction. All PET and CT data were processed using OsiriX Medical Imaging software (v.13.0.3) as described before^53^.

### RNA isolation and RT-qPCR

Bone-marrow-derived macrophages cultured in RPMI 1640 medium containing sodium pyruvate (1% P/S, 15% L929 conditioned medium) were incubated for 24 h with aNP-siRNA. The NucleoSpin kit (Machery Nagel) was used to isolate RNA from 750,000 BMDMs 48 h after incubation. The RNA was reverse transcribed using the iScript cDNA Synthesis Kit (Biorad) at 300 ng per reaction. The resulting cDNA was diluted tenfold and amplified in a PowerUp SYBR Green mastermix (Applied Biosystems) using a QuantStudio 3 (Applied Biosystems) thermocycler. A final concentration of 100 nM for the reverse and forward primer was used (primer (IDT) details are listed in Supplementary Table 2). Thermocycling was performed for 2 min at 95 °C followed by forty 15 s amplification cycles at 95 °C, and a further forty 30 s amplification cycles at 60 °C, with fluorescence acquisition at the end of each cycle. Reactions were performd in duplicate and each plate included an NTC and a reverse transcriptase control. To verify the amplification of the intended product a melting curve analysis was performed from 65 °C to 95 °C. All amplification data were normalized to the ROX dye before further processing. Each gene of interest was normalized to two housekeeping genes and RT-qPCR, whereas the ΔCq-expression was normalized to aNP-siCtrl at an equivalent concentration. The percentage knockdown is calculated according to the following formula: %KD = (1 − ∆∆Cq) × 100.

### Preparing single-cell suspensions and staining for flow cytometry

Single-cell suspensions comprised whole blood, spleen and bone marrow tissue retrieved from euthanized female C57BL/6J mice. The entire spleen was directly minced on a Corning 70 µm cell strainer using a syringe plunger and flow buffer (DPBS, 0.5% BSA, 2 mM EDTA). Single-cell suspensions of the bone marrow were made by flushing the femurs with 10 ml flow buffer. The bone marrow was then immediately filtered using a 70 µm cell strainer and centrifugated at 400 × *g* for 5 min at 4 °C, aspirating the supernatant. The cell pellets from the whole blood, spleen and bone marrow were resuspended in 1× red blood cell lysis (catalogue no. 420302, Biolegend). The samples were then incubated for 3 × 7 min on ice (blood), or at room temperature for 4 min (spleen) or 1 min (bone marrow). Quenching was achieved with flow buffer and cells were centrifuged at 400 × *g* for 5 min at 4 °C. Next, the single cells were transferred to a Corning V-bottom 96-well plate and incubated with 50 µl viakrome 808 (catalogue no. C36628, Beckman Coulter) diluted in PBS for 20 min in the dark at room temperature. The cells were then washed, centrifuged again and incubated with CD16/32 (Fc-block, 101302, BioLegend) antibodies (except for the progenitor staining group) for 10 min on ice. The cells were washed, centrifuged, and brilliant stain buffer (Invitrogen) containing antibodies were added (total volume of 50 µl). The sample was then incubated on ice for 30 min in the dark. All data were acquired using a 21-colour CytoFLEX LX (Beckman Coulter). The antibodies used for the myeloid cells panel and progenitor cells panel are listed in Supplementary Tables 5–8.

### Clinical chemistry and cytokine measurements

Female C57BL/6J mice were intravenously administrated with 0.5 mg kg^–1^ aNP-siRNA or PBS (*n* = 4 per group). After 4 h, serum was collected, and cytokine (IL-6 and TNF-α) levels were measured using enzyme-linked immunosorbent assay (ELISA) kits (ELISA MAX Deluxe Set Mouse IL-6 (Biolegend) and ELISA MAX Deluxe Set Mouse TNF-α, Biolegend) according to the manufacturer’s instructions. After 24 h and 72 h, whole blood was collected in BD Vacutainer Heparin Tubes (Becton Dickinson) and the serum was separated. The liver function (ASAT and ALAT) and renal function (urea and creatinine) were measured at the Radboudumc Laboratorium voor Diagnostiek core facility.

### Liver histology

Mice were euthanized and perfused with PBS. Whole livers were collected and fixed in 10% formalin for 24 h. The tissue was subsequently processed and embedded in paraffin wax at the diagnostic pathology department. Paraffin sections (5 µm thickness) were cut using a Leica RM2235 microtome, mounted on Superfrost Plus Microscope slides (VWR), and stained with haematoxylin and eosin. The slides were scanned and visualized using CaseViewer software (3DHISTECH).

### Inbred mice strain

All animal experiments were conducted in compliance with European Union and Dutch guidelines according to the care and use of laboratory mice after approval by the Radboud University Medical Center’s Dierexperimentencommissie (DEC) (CCD License = AVD10300 2021 15550); 8–12 weeks old female C57BL/6J mice (*Mus musculus*) obtained from Charles River were co-housed in climate-controlled conditions (20–24 °C and a humidity level of 45–65% RV). An acclimatization period of one week was used and mice had access to food and water ad libitum. Mice were randomized and assigned to control and treatment groups. Studies were conducted in a blinded manner.

### Tumour inoculation and treatment regimen

Female C57BL/6J mice were inoculated with 5 × 10^5^ MC38 cells in 100 µl PBS via subcutaneous injection in the right flank on day –10. Treatment for all experiments consisted of intravenous administration (7 × 0.5 mg kg^–1^ in 14 days, 48 h apart) of aNP_18_-siCtrl or aNP_18_-siCCR2. Treatment started on day 0 and lasted until day 14. The study was stopped at day 14 while tumour volumes were <2,000 mm^3^ (humane endpoint) to comply with Dutch regulations on animal welfare. Minimum group sizes were determined by statistical power calculations (Piface) while keeping rates for type I and II errors <5% and a power of 80%.

### Statistical analysis

All data are presented as mean ± s.d. or standard error of mean and analysed using GraphPad Prism v.10.0 by one-way ANOVA or Student's *t*-test, as detailed in the figure captions. *P*-values < 0.05 were considered significant, with levels of significance indicated as: *P < 0.05; **P < 0.01; ***P < 0.001; ****P < 0.0001. Data distribution was assumed to be normal but this was not formally tested.

### Figure design

Schematic figures were prepared using BioRender (BioRender.com) and Servier Medical Art (smart.servier.com).

### Reporting summary

Further information on research design is available in the Nature Portfolio Reporting Summary linked to this article.

## Online content

Any methods, additional references, Nature Portfolio reporting summaries, source data, extended data, supplementary information, acknowledgements, peer review information; details of author contributions and competing interests; and statements of data and code availability are available at 10.1038/s41565-024-01847-3.

## Supplementary information

Supplementary Information Supplementary Figs. 1–5 and Tables 1–8.

Reporting Summary

Supplementary Data Supplementary Fig. 1 source data.

## Source data

Source Data Fig. 1 Numerical and/or statistical source data.

Source Data Fig. 2 Numerical and/or statistical source data.

Source Data Fig. 3 Numerical and/or statistical source data.

Source Data Fig. 4 Numerical and/or statistical source data.

Source Data Fig. 5 Numerical and/or statistical source data.

Source Data Extended Data Fig. 3 Numerical and/or statistical source data.

Source Data Extended Data Fig. 4 Numerical and/or statistical source data.

Source Data Extended Data Fig. 5 Numerical and/or statistical source data.

Source Data Extended Data Fig. 6 Numerical and/or statistical source data.

Source Data Extended Data Fig. 7 Numerical and/or statistical source data.

Source Data Extended Data Fig. 8 Numerical and/or statistical source data.

Source Data Extended Data Fig. 9 Numerical and/or statistical source data.

Source Data Extended Data Fig. 10 Numerical and/or statistical source data.

## Data Availability

The data supporting the results in this study are available within the main text, the Extended Data and the Supplementary Information. The Source Data of the cryo-EM analysis and liver histology are available for research purposes from the corresponding authors on reasonable request. Source Data are provided with this paper.
