# Supplementary material for: Nature-inspired platform nanotechnology for RNA delivery to myeloid cells and their bone marrow progenitors
Source: Nat Nanotechnol. 2025 Feb 3;20(4):532–42. doi: 10.1038/s41565-024-01847-3 (PMC12014499; doi:10.1038/s41565-024-01847-3)
Supplement: Supplementary file 1 — Supplementary Figs. 1–5 and Tables 1–8. [file 41565_2024_1847_MOESM1_ESM.pdf]

nature nanotechnology

Supplementary information https://doi.org/10.1038/s41565-024-01847-3

Nature-inspired platform nanotechnology for RNA delivery to myeloid cells and their bone marrow progenitors

In the format provided by the authors and unedited

SUPPLEMENTARY INFORMATION

TABLE OF CONTENTS

SUPPLEMENTARY FIGURES	Page
Supplementary Figure 1	2
Supplementary Figure 2	3
Supplementary Figure 3	9
Supplementary Figure 4	10
Supplementary Figure 5	11
**SUPPLEMENTARY TABLES**	
Supplementary Table 1	12
Supplementary Table 2	13
Supplementary Table 3	13
Supplementary Table 4	13
Supplementary Table 5	14
Supplementary Table 6	14
Supplementary Table 7	15
Supplementary Table 8	15

SUPPLEMENTARY FIGURES

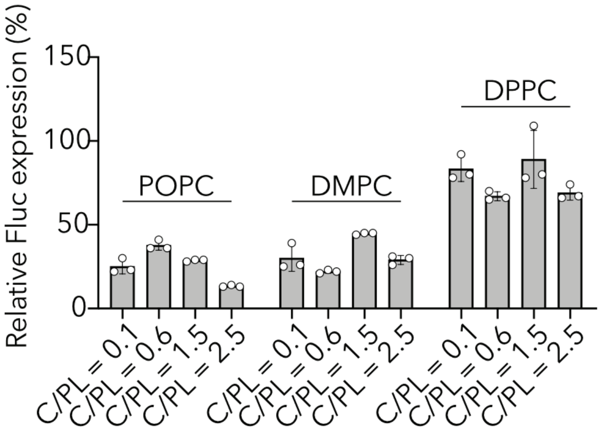

Supplementary Figure 1. Reporter gene silencing *in vitro* for aNP formulations with diverse compositions.
Normalized firefly luciferase knockdown in dual-luciferase reporter RAW264.7 cells of aNP formulations containing firefly luciferase siRNA with a cholesterol (C) to phospholipid (PL) ratio ranging from 0.1 to 2.5 and containing phospholipid types POPC, DMPC, or DPPC. Data represent mean ± SD of one experiment with three technical repeats (n=3).

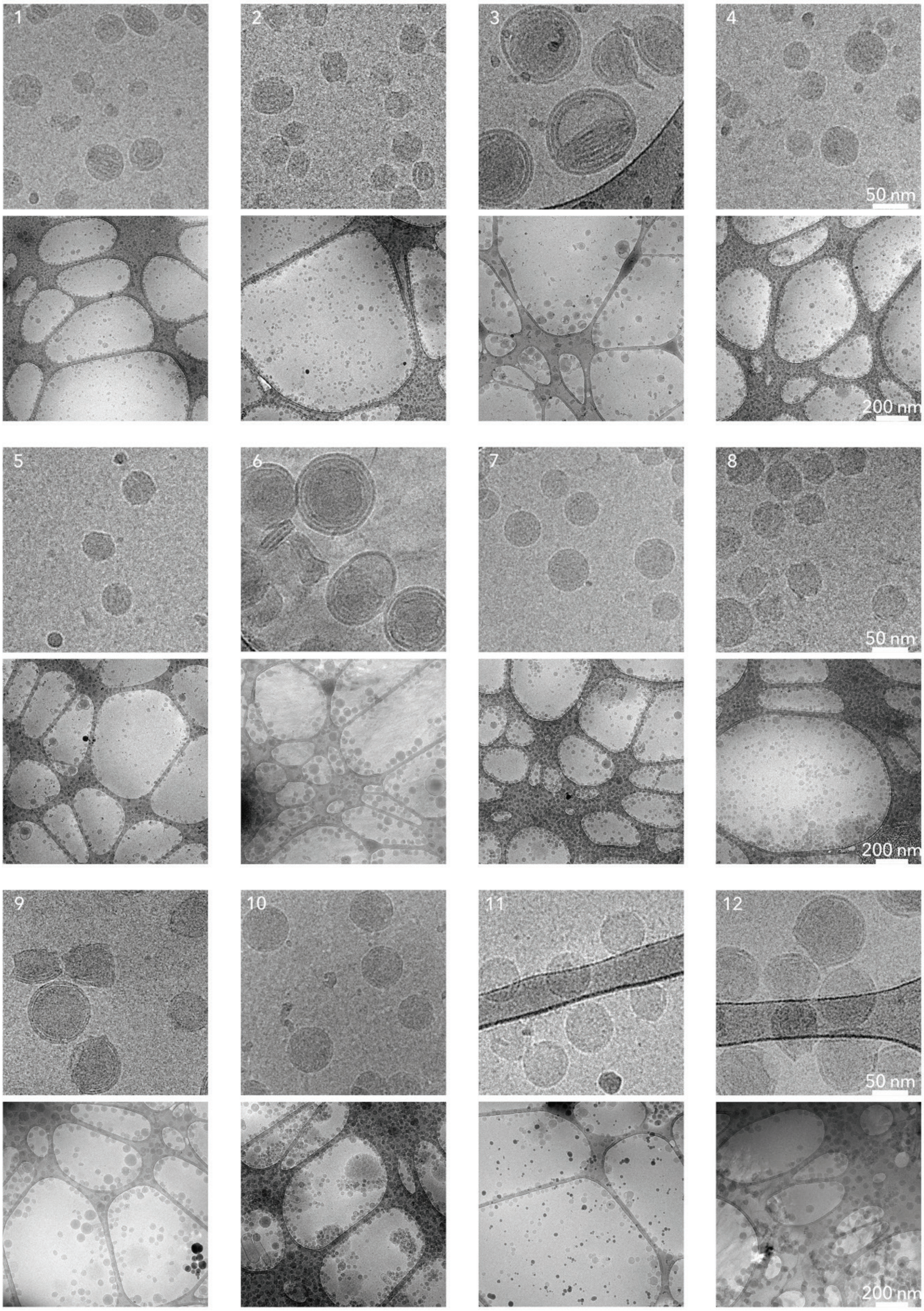

Supplementary Figure 2 continues on the next page.

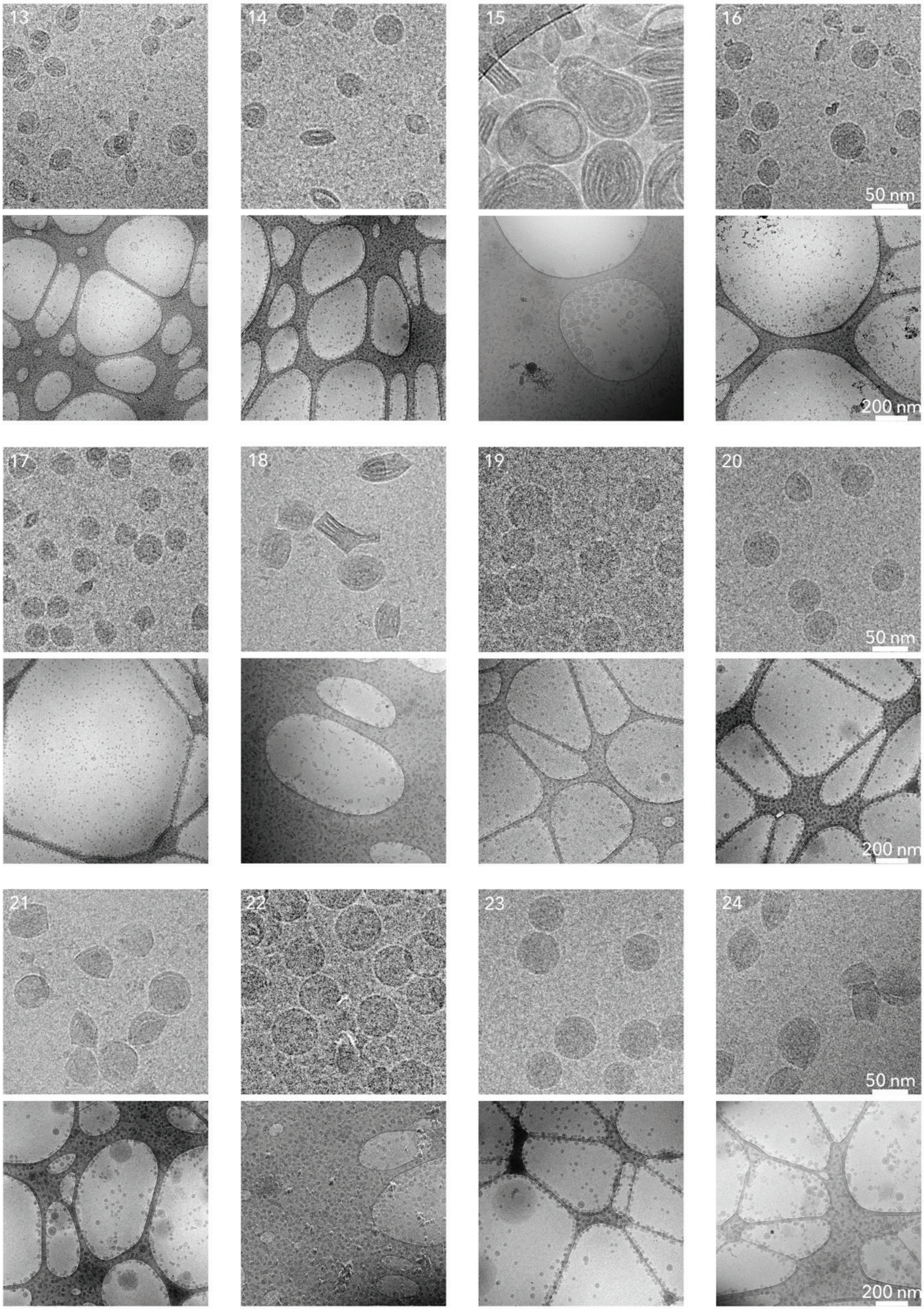

Supplementary Figure 2 continues on the next page.

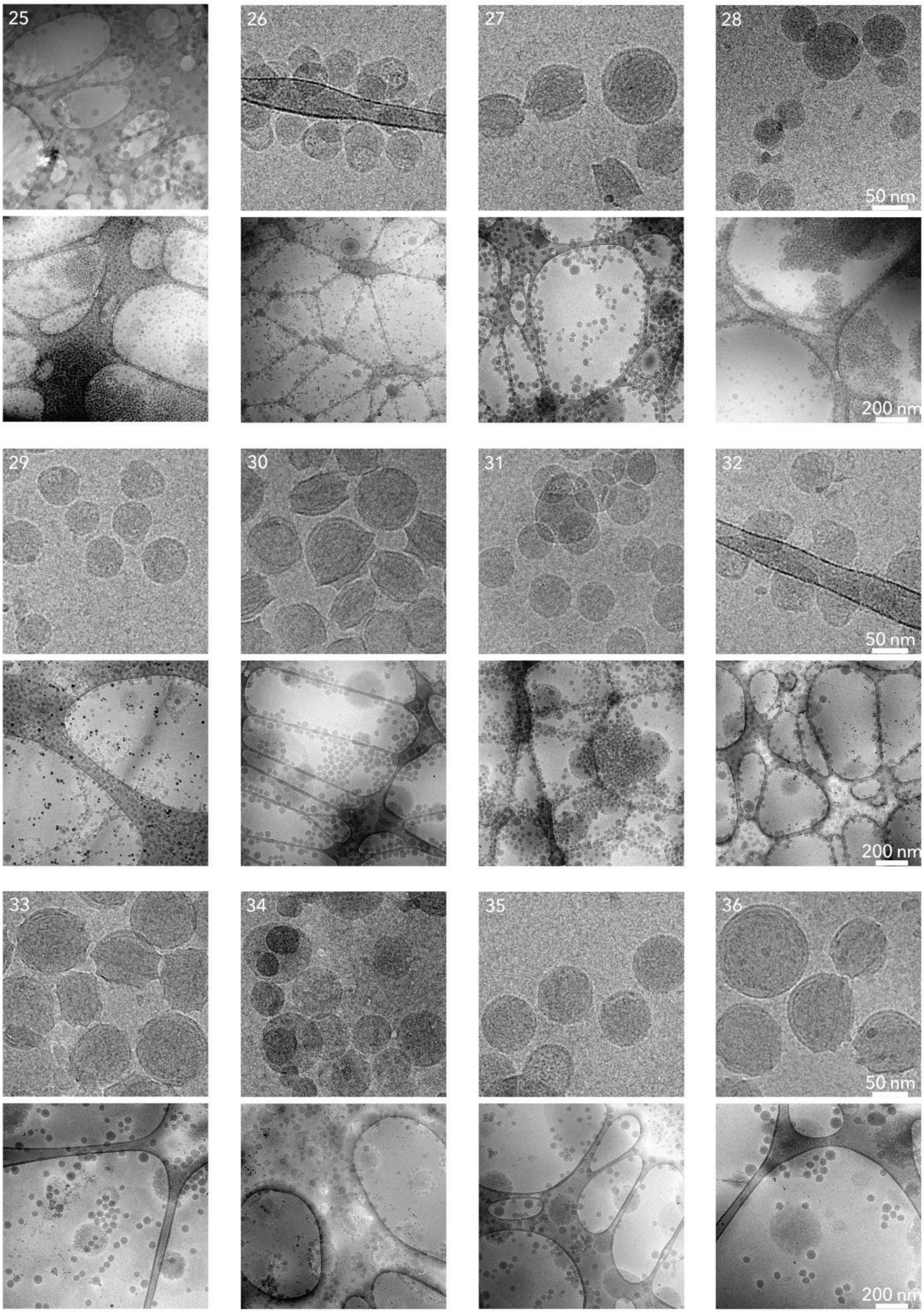

Supplementary Figure 2 continues on the next page.

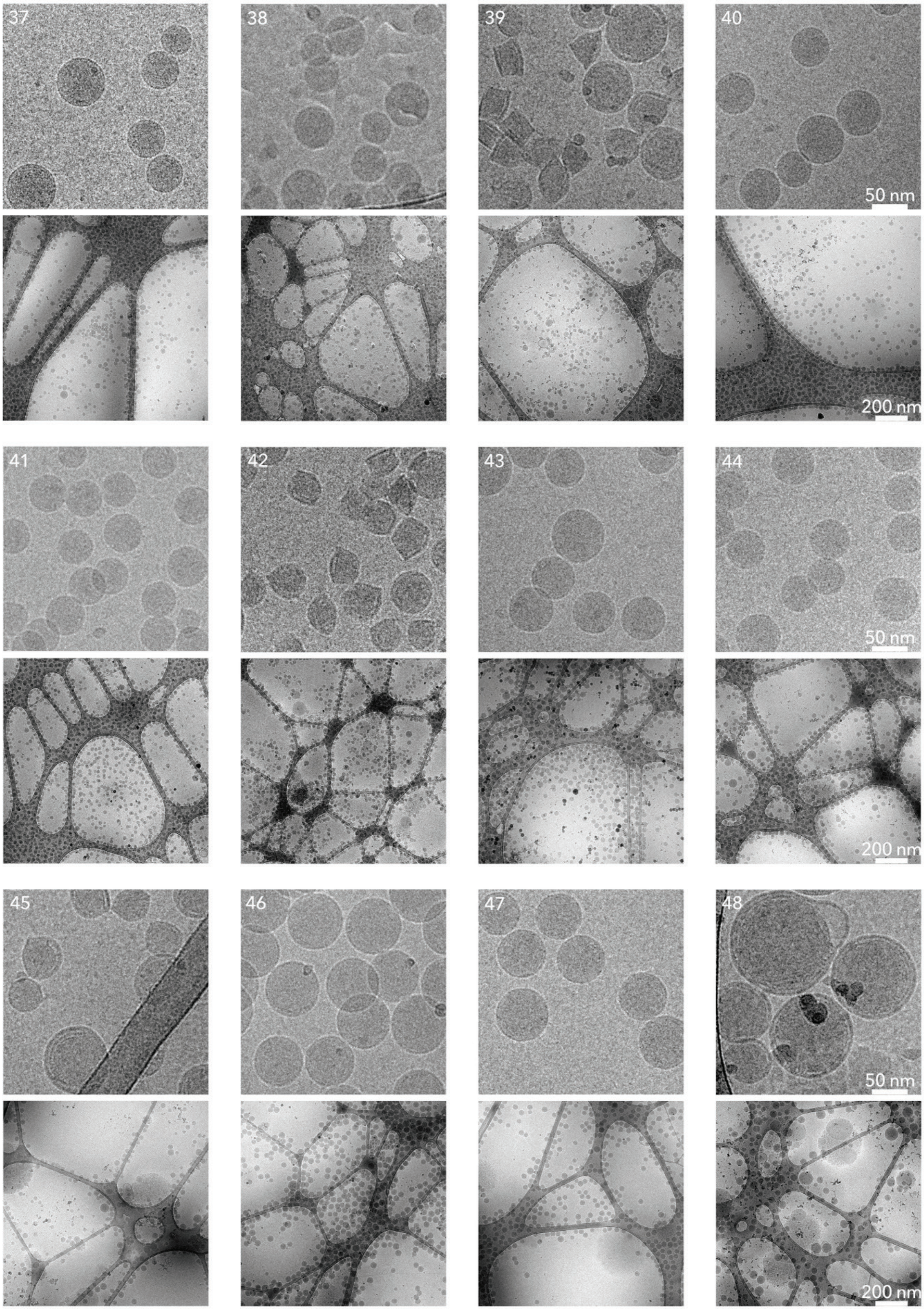

Supplementary Figure 2 continues on the next page.

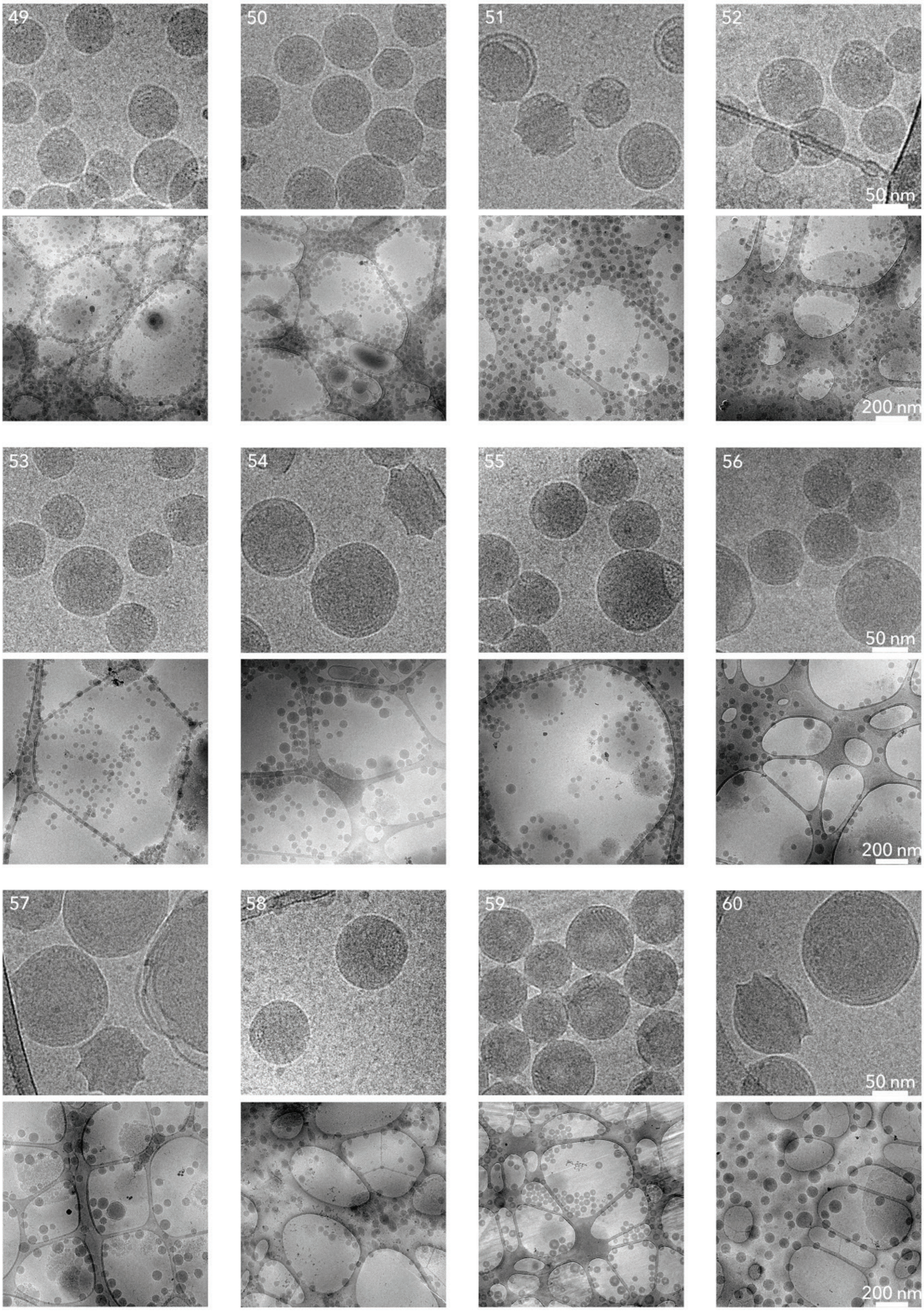

Supplementary Figure 2 continues on the next page.

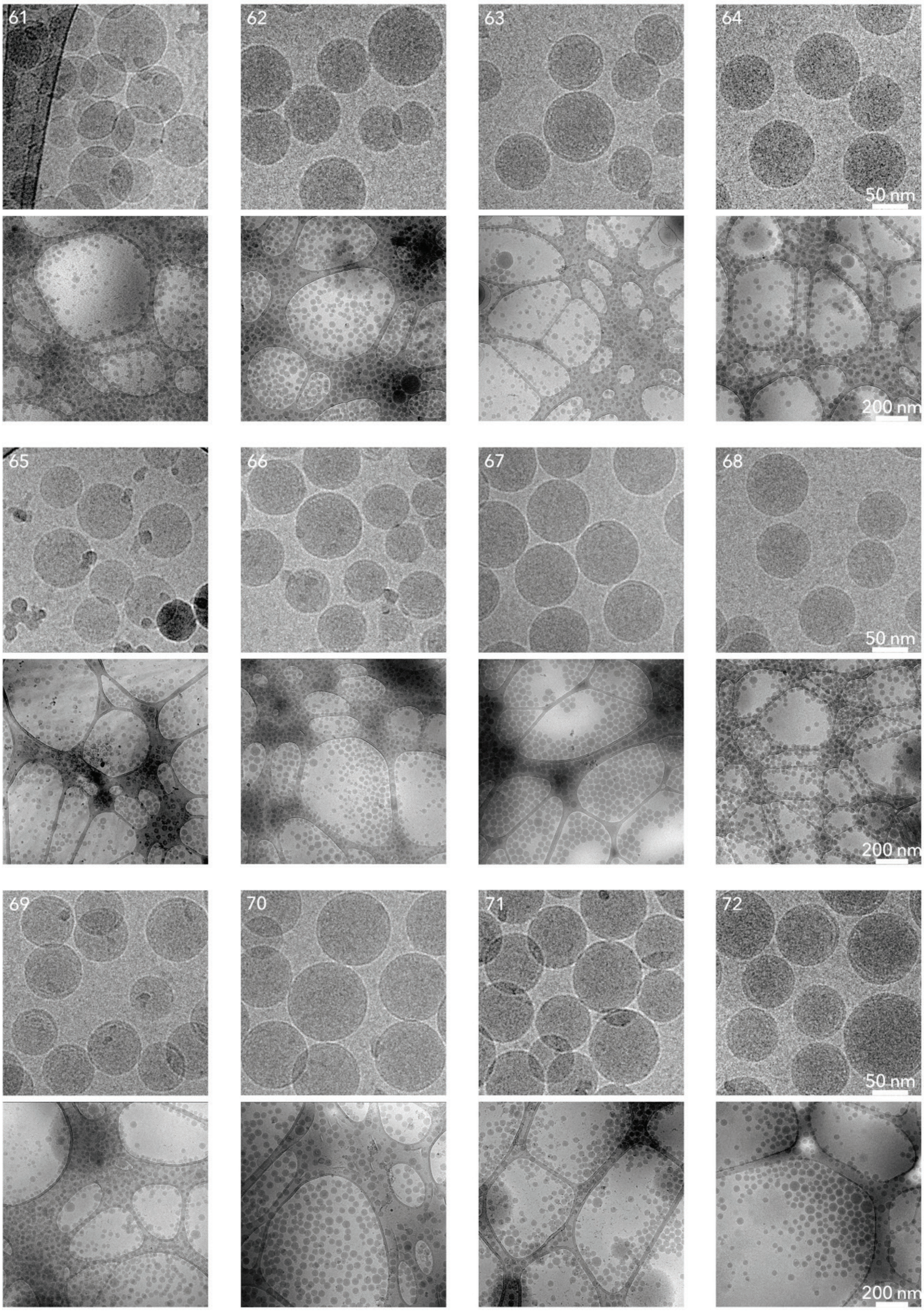

Supplementary Figure 2. Comprehensive aNP-siRNA library cryo-EM overview.
Detailed cryo-EM images of 72 aNP-siRNA formulations indicated by numerical identifier. **Top panels.** Cryo-EM image at a 24000-fold magnification. The scalebar represents 50 nm. **Bottom panels.** Higher level overview of the formulation at a 6500-fold magnification. Scale bar represents 200 nm.

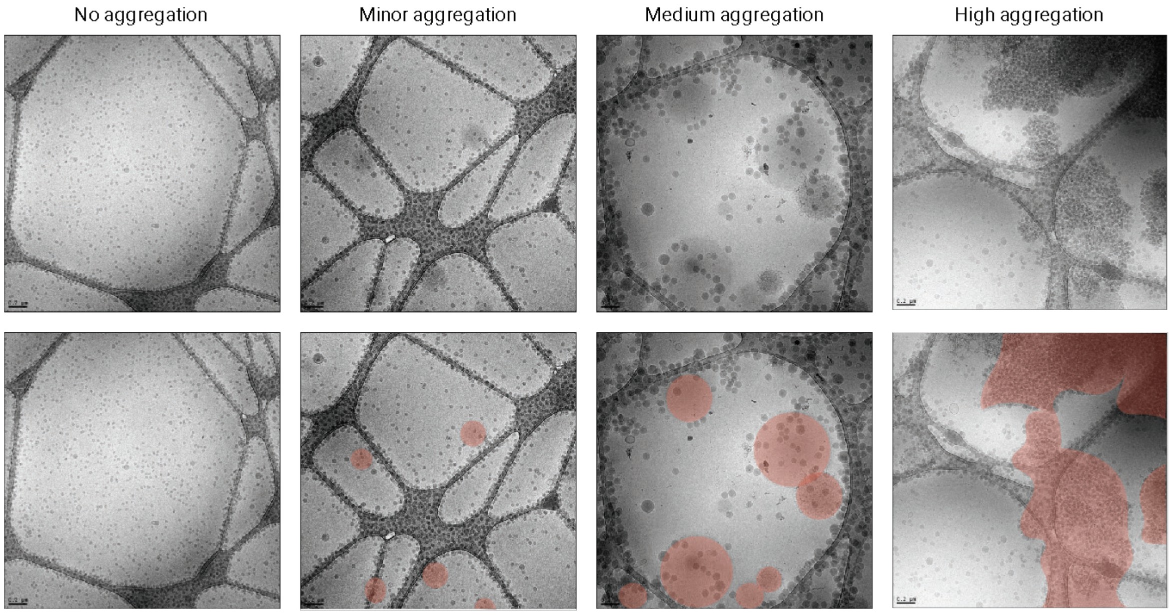

Supplementary Figure 3. Representative cryo-EM images displaying no aggregation to high aggregation.
The degree of aggregation was assessed by determining the area of lipid aggregates (masked in red) divided by the total area of the image (n=10).

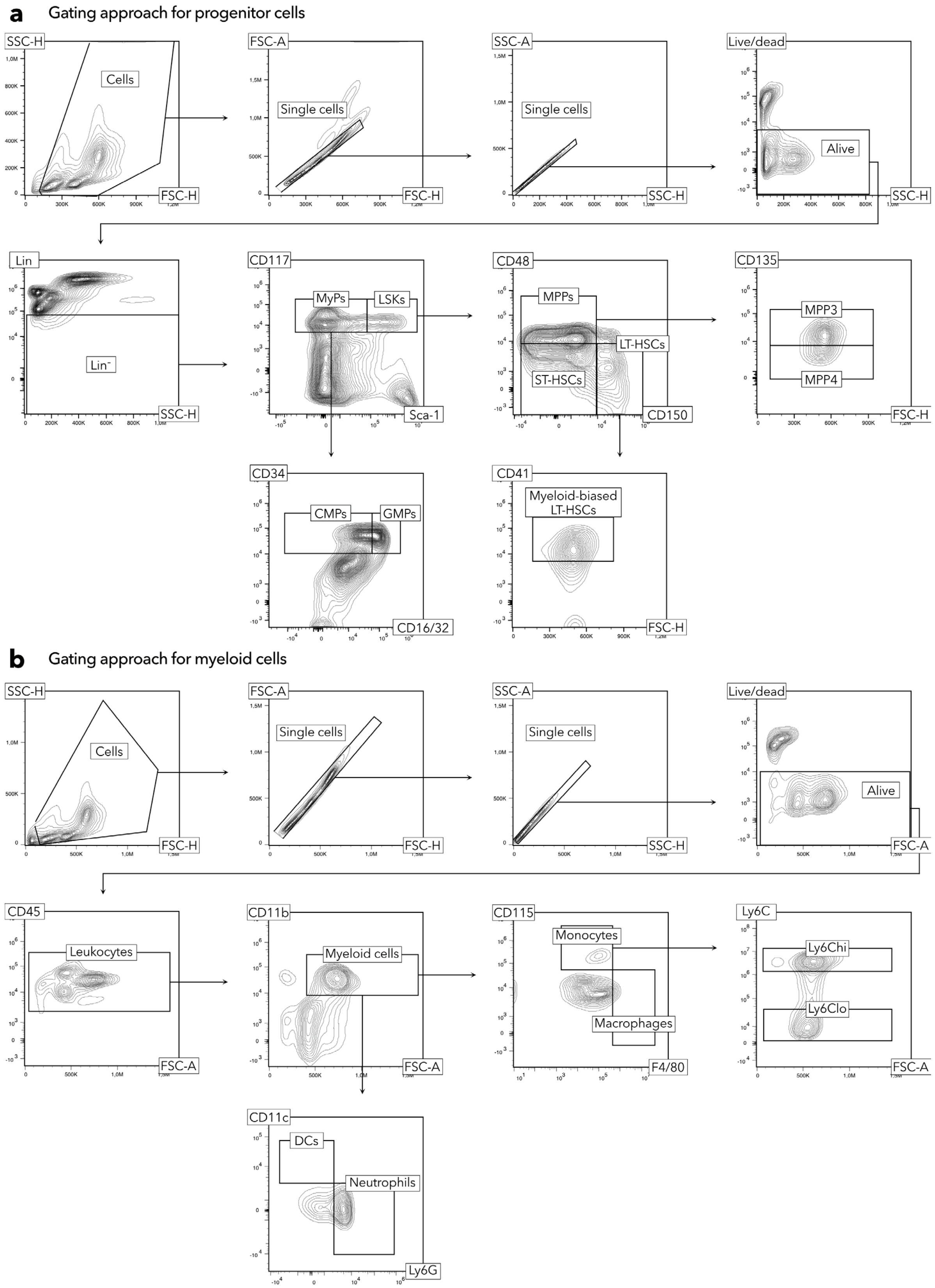

Supplementary Figure 4. Gating strategy for flow cytometry analysis.
a. Progenitor cells**. b.** Myeloid cells. Antibody panels are listed in the **Supplementary Table 5-8**.

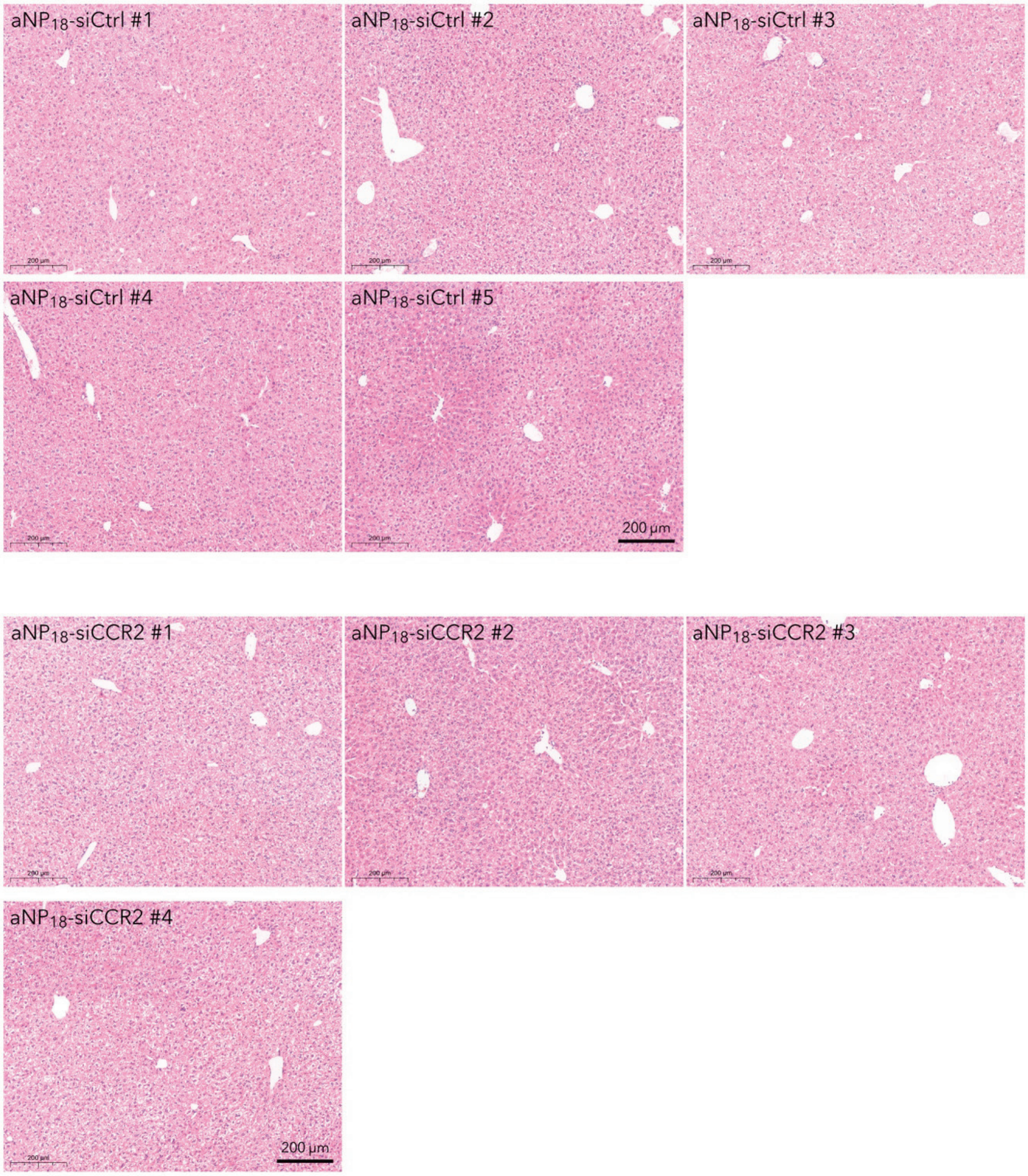

Supplementary Figure 5. Liver histology.
Paraffin-fixated liver sections (5 μm thickness) were stained with hematoxylin and eosin. Shown are five liver sections from mice treated with aNP_{18}-siCtrl (**top**), and four liver sections from mice treated with aNP_{18}-siCCR2 (**bottom**). Scale bar represents 200 μm.

SUPPLEMENTARY TABLES

Supplementary Table 1. Compositions of aNP-siRNA library formulations.

	Phospholipid			Dlin-MC3-DMA		Cholesterol		Triglycerides		ApoA1		siRNA	
Formulation#	Type	mg	mol%	mg	mol%	mg	mol%	mg	mol%	mg	mol%	mg	mol%
1	POPC	2.5	49%	1.25	29%	0.54	21%	0	0%	1	0.50%	0.2	0.18%
4	POPC	2.5	38%	2.5	45%	0.54	16%	0	0%	1	0.39%	0.2	0.14%
7	POPC	2.5	31%	3.75	55%	0.54	13%	0	0%	1	0.32%	0.2	0.11%
10	POPC	2.5	26%	5	62%	0.54	11%	0	0%	1	0.27%	0.2	0.10%
13	DMPC	2.5	52%	1.25	28%	0.54	20%	0	0%	1	0.47%	0.2	0.17%
16	DMPC	2.5	41%	2.5	43%	0.54	15%	0	0%	1	0.37%	0.2	0.13%
19	DMPC	2.5	34%	3.75	53%	0.54	13%	0	0%	1	0.31%	0.2	0.11%
22	DMPC	2.5	29%	5	60%	0.54	11%	0	0%	1	0.26%	0.2	0.09%
2	POPC	2.5	37%	1.25	22%	1.35	40%	0	0%	1	0.38%	0.2	0.14%
5	POPC	2.5	31%	2.5	36%	1.35	33%	0	0%	1	0.31%	0.2	0.11%
8	POPC	2.5	26%	3.75	46%	1.35	28%	0	0%	1	0.26%	0.2	0.10%
11	POPC	2.5	22%	5	53%	1.35	24%	0	0%	1	0.23%	0.2	0.08%
14	DMPC	2.5	40%	1.25	21%	1.35	38%	0	0%	1	0.37%	0.2	0.13%
17	DMPC	2.5	33%	2.5	35%	1.35	31%	0	0%	1	0.30%	0.2	0.11%
20	DMPC	2.5	28%	3.75	45%	1.35	27%	0	0%	1	0.26%	0.2	0.09%
23	DMPC	2.5	25%	5	52%	1.35	23%	0	0%	1	0.22%	0.2	0.08%
3	POPC	2.5	23%	1.25	14%	3.37	62%	0	0%	1	0.24%	0.2	0.09%
6	POPC	2.5	21%	2.5	24%	3.37	55%	0	0%	1	0.21%	0.2	0.08%
9	POPC	2.5	18%	3.75	33%	3.37	49%	0	0%	1	0.19%	0.2	0.07%
12	POPC	2.5	17%	5	39%	3.37	44%	0	0%	1	0.17%	0.2	0.06%
15	DMPC	2.5	26%	1.25	14%	3.37	61%	0	0%	1	0.23%	0.2	0.08%
18	DMPC	2.5	23%	2.5	24%	3.37	53%	0	0%	1	0.21%	0.2	0.07%
21	DMPC	2.5	20%	3.75	32%	3.37	48%	0	0%	1	0.18%	0.2	0.07%
24	DMPC	2.5	18%	5	38%	3.37	43%	0	0%	1	0.17%	0.2	0.06%
25	POPC	2.5	19%	1.25	11%	0.54	8%	5	61%	1	0.19%	0.2	0.07%
28	POPC	2.5	17%	2.5	20%	0.54	7%	5	55%	1	0.17%	0.2	0.06%
31	POPC	2.5	16%	3.75	28%	0.54	7%	5	50%	1	0.16%	0.2	0.06%
34	POPC	2.5	14%	5	34%	0.54	6%	5	46%	1	0.14%	0.2	0.05%
37	DMPC	2.5	21%	1.25	11%	0.54	8%	5	60%	1	0.19%	0.2	0.07%
40	DMPC	2.5	19%	2.5	20%	0.54	7%	5	54%	1	0.17%	0.2	0.06%
43	DMPC	2.5	17%	3.75	27%	0.54	6%	5	49%	1	0.16%	0.2	0.06%
46	DMPC	2.5	16%	5	33%	0.54	6%	5	45%	1	0.14%	0.2	0.05%
26	POPC	2.5	17%	1.25	10%	1.35	18%	5	55%	1	0.17%	0.2	0.06%
29	POPC	2.5	15%	2.5	18%	1.35	16%	5	50%	1	0.16%	0.2	0.06%
32	POPC	2.5	14%	3.75	25%	1.35	15%	5	46%	1	0.14%	0.2	0.05%
35	POPC	2.5	13%	5	31%	1.35	14%	5	42%	1	0.13%	0.2	0.05%
38	DMPC	2.5	19%	1.25	10%	1.35	18%	5	54%	1	0.17%	0.2	0.06%
41	DMPC	2.5	17%	2.5	18%	1.35	16%	5	49%	1	0.15%	0.2	0.06%
44	DMPC	2.5	16%	3.75	25%	1.35	15%	5	45%	1	0.14%	0.2	0.05%
47	DMPC	2.5	14%	5	30%	1.35	14%	5	41%	1	0.13%	0.2	0.05%
27	POPC	2.5	13%	1.25	8%	3.37	35%	5	43%	1	0.14%	0.2	0.05%
30	POPC	2.5	12%	2.5	15%	3.37	33%	5	40%	1	0.13%	0.2	0.05%
33	POPC	2.5	12%	3.75	20%	3.37	31%	5	37%	1	0.12%	0.2	0.04%
36	POPC	2.5	11%	5	26%	3.37	29%	5	35%	1	0.11%	0.2	0.04%
39	DMPC	2.5	15%	1.25	8%	3.37	35%	5	42%	1	0.13%	0.2	0.05%
42	DMPC	2.5	14%	2.5	14%	3.37	32%	5	39%	1	0.12%	0.2	0.04%
45	DMPC	2.5	13%	3.75	20%	3.37	30%	5	37%	1	0.12%	0.2	0.04%
48	DMPC	2.5	12%	5	25%	3.37	28%	5	34%	1	0.11%	0.2	0.04%
49	POPC	2.5	12%	1.25	7%	0.54	5%	10	76%	1	0.12%	0.2	0.04%
52	POPC	2.5	11%	2.5	13%	0.54	5%	10	71%	1	0.11%	0.2	0.04%
55	POPC	2.5	10%	3.75	18%	0.54	4%	10	67%	1	0.11%	0.2	0.04%
58	POPC	2.5	10%	5	23%	0.54	4%	10	63%	1	0.10%	0.2	0.04%
61	DMPC	2.5	13%	1.25	7%	0.54	5%	10	75%	1	0.12%	0.2	0.04%
64	DMPC	2.5	12%	2.5	13%	0.54	5%	10	70%	1	0.11%	0.2	0.04%
67	DMPC	2.5	11%	3.75	18%	0.54	4%	10	66%	1	0.10%	0.2	0.04%
70	DMPC	2.5	11%	5	23%	0.54	4%	10	62%	1	0.10%	0.2	0.04%
50	POPC	2.5	11%	1.25	6%	1.35	12%	10	71%	1	0.11%	0.2	0.04%
53	POPC	2.5	10%	2.5	12%	1.35	11%	10	66%	1	0.10%	0.2	0.04%
56	POPC	2.5	10%	3.75	17%	1.35	10%	10	63%	1	0.10%	0.2	0.04%
59	POPC	2.5	9%	5	22%	1.35	10%	10	59%	1	0.09%	0.2	0.03%
62	DMPC	2.5	12%	1.25	6%	1.35	11%	10	70%	1	0.11%	0.2	0.04%
65	DMPC	2.5	11%	2.5	12%	1.35	11%	10	66%	1	0.10%	0.2	0.04%
68	DMPC	2.5	11%	3.75	17%	1.35	10%	10	62%	1	0.10%	0.2	0.04%
71	DMPC	2.5	10%	5	21%	1.35	10%	10	59%	1	0.09%	0.2	0.03%
51	POPC	2.5	9%	1.25	6%	3.37	25%	10	60%	1	0.10%	0.2	0.03%
54	POPC	2.5	9%	2.5	10%	3.37	23%	10	57%	1	0.09%	0.2	0.03%
57	POPC	2.5	8%	3.75	15%	3.37	22%	10	54%	1	0.09%	0.2	0.03%
60	POPC	2.5	8%	5	19%	3.37	21%	10	52%	1	0.08%	0.2	0.03%
63	DMPC	2.5	10%	1.25	5%	3.37	24%	10	60%	1	0.09%	0.2	0.03%
66	DMPC	2.5	10%	2.5	10%	3.37	23%	10	57%	1	0.09%	0.2	0.03%
69	DMPC	2.5	9%	3.75	15%	3.37	22%	10	54%	1	0.08%	0.2	0.03%
72	DMPC	2.5	9%	5	19%	3.37	21%	10	51%	1	0.08%	0.2	0.03%
LNP	DSPC	0.31	10%	1.25	50%	0.58	38.5%	PEG-DMG:	1.5%	0.145 mg		0.2	
aNP-mRNA	DMPC	2.5	17%	ALC0315: 2.8	17%	1.4	17%	5	49%	1.1		mRNA	0.2 mg
aNP-ASO	POPC	2	25%	ALC0315: 4	50%	1	25%	0		0.8		ASO	0.2 mg
LNP-mRNA	DSPC	0.6	10%	ALC0315: 2.8	50%	1.1	38.5%	PEG-DMG:	1.5%	0.28 mg		mRNA	0.2 mg

Supplementary Table 2. Cryo-EM recorded sizes of aNP-siRNA in the tested series of formulations.

Formulation	Diameter (nm)	Formulation	Diameter (nm)	Formulation	Diameter (nm)	Formulation	Diameter (nm)
1	37 ± 10	19	53 ± 5	37	52 ± 9	55	82 ± 13
2	46 ± 8	20	42 ± 5	38	47 ± 7	56	88 ± 33
3	100 ± 26	21	57 ± 9	39	60 ± 11	57	110 ± 22
4	40 ± 9	22	65 ± 7	40	58 ± 7	58	88 ± 12
5	51 ± 8	23	51 ± 6	41	49 ± 7	59	84 ± 20
6	93 ± 24	24	65 ± 20	42	51 ± 9	60	125 ± 28.3
7	48 ± 11	25	44 ± 9	43	66 ± 6	61	70 ± 11
8	54 ±14	26	57 ± 13	44	59 ± 8	62	73 ± 14
9	80 ± 17	27	81 ± 19	45	61 ± 11	63	63 ± 13
10	51 ± 12	28	51 ± 13	46	77 ± 10	64	78 ± 9
11	69 ± 19	29	58 ± 11	47	68 ± 9	65	65 ± 9
12	118 ± 40	30	80 ± 11	48	87 ± 28	66	62 ± 12
13	32 ± 7	31	59 ± 11	49	66 ± 12	67	83 ± 10
14	37 ± 7	32	70 ± 11	50	68 ± 13	68	70 ± 8
15	77 ± 23	33	95 ± 13	51	91 ± 21	69	65 ± 13
16	36 ± 6	34	77 ± 11	52	70 ± 10	70	90 ± 11
17	36 ± 6	35	81 ± 20	53	71 ± 11	71	82 ± 15
18	18 ± 11	36	108 ± 15	54	98 ± 17	72	84 ± 16

Supplementary Table 3. Primer sets for target and housekeeping genes used in RT-qPCR.

Target	Forward (5'- 3')	Reverse (5'- 3')	qPCR efficiency (%)
LAMP1	AACAACGGAACCTGCCTGCT	GGGCTGATGTTGAACGCTCTG	92
CCR2	TGCCATCATAAAGGAGCCATACC	TGCCGTGGATGAACTGAGGT	110
RPL4	CTTCGCCAGGCCAGAAATCA	TCTCGGATTTGGTTGCCAGTG	102
b2M	TTCTGGTGCTTGTCTCACTGA	CAGTATGTTCGGCTTCCCATTC	94

Supplementary Table 4. siRNA sequences.

Target	Sense (5'- 3')	Antisense (5'- 3')
siFLuc a	rGrGrUrGrCrCrCrGrGrCrArCrCrArUrCrGrCrCrUrUrUrAdCdC	rCrArCrArGrGrUrUrCrArGrGrUrGrGrUrGrGrArArUrCrGrGrArGrC
siFLuc b	rGrCrArCrArUrArUrCrGrArGrGrUrGrGrArCrArUrUrArCdCdT	rGrCrCrArCrArGrGrUrUrCrArGrGrUrGrGrUrGrGrArArUrCrGrGrA
siFLuc c	rArUrCrGrArGrGrUrGrGrArCrArUrUrArCrCrUrArCrGrCdCdG	rGrUrGrCrArArArArGrArArGrCrUrArCrCrGrArUrCrArUrArCrArA
siFLuc d	rGrArGrGrCrUrArArGrGrUrGrGrUrGrGrArCrUrUrGrGrAdCdA	rArArArCrArUrArCrUrArGrCrCrArUrCrGrArArGrArArArArCrGrU
siFLuc e	rGrCrArArArArGrArArGrCrUrArCrCrGrArUrCrArUrArCdAdA	rArCrArGrGrUrUrCrArGrGrUrGrGrUrGrGrArArUrCrGrGrArGrCrU
*siFLuc f	rGrCrArArArArGrArArGrCrUrArCrCrGrArUrCrArUrArCdAdA	rGrCrCrGrCrArUrCrCrArUrUrArCrArGrGrUrGrGrArGrCrUrArUrA
siFLuc g	rGrCrUrArArGrGrUrGrGrUrGrGrArCrUrUrGrGrArCrArCdCdG	rUrCrCrArUrUrArCrArGrGrUrGrGrArGrCrUrArUrArCrArCrGrCrA
siFLuc h	rArGrGrCrUrArArGrGrUrGrGrUrGrGrArCrUrUrGrGrArCdAdC	rCrCrArUrUrUrCrCrGrCrUrArCrCrArCrGrGrCrCrCGrUrGrGrUrC
siLAMP1a	mCmCrCmArCmArUrUrCrArGmCrAmUrCmUrCmCrArArCrCrAmUdT	rArAmUrGrGrUrUrGmGrAmGrAmUrGrCrUrGrArArUrGrUmGrGmGmAmC
siLAMP1b	mGmGrAmUrAmUrUrUrArCrUmGrAmCrAmCrUmCrArArCrUrUmCdA	rUrGmArArGrUrUrGmArGmUrGmUrCrArGrUrArArArUrAmUrCmCmUmC
siLAMP1c	mGmGrAmUrAmUrUrUrArCrUmGrAmCrAmCrUmCrArArCrUrUmCdA	rUrUmGrArGrUrGrUmCrAmGrUmArArArUrArUrCrCrUrCmUrUmCmCmA
siLAMP1d	mUmGrGmCrCmUrCrUrArUrGmGrCmArCmUrGmCrArArCrUrGmAdA	rUrUmCrArGrUrUrGmCrAmGrUmGrCrCrArUrArGrArGrGmCrCmAmGmC
*siLAMP1e	/5Phos/mGmArAmGrAmGrGrArUrArUmUrUmArCmUrGmArCrArCrUrCmAdA	rUrUmGrArGrUrGrUmCrAmGrUmArArArUrArUrCrCrUrCmUrUmCmCmA
siLAMP1f	mAmArGmUrUmCrArUrCrUrUmGrCmArAmCrAmUrUrUrArCrUmAdT	rArUmArGrUrArArAmUrGmUrUmGrCrArArGrArUrGrArAmCrUmUmCmA
siLAMP1g	mGmCrAmCrUmGrCrArArCrUmGrAmArUmArUmCrArCrCrUrAmCdC	rGrGmUrArGrGrUrGmArUmArUmUrCrArGrUrUrGrCrArGmUrGmCmCmA
siLAMP1h	mCmArAmUrUmArCrUrUrUrUmGrGmArAmGrAmGrGrArUrArUmUdT	rArAmArUrArUrCrCmUrCmUrUmCrCrArArArArGrUrArAmUrUmGmUmG
siCCR2a	rGrUrUrCrCrUrUrArCrArUrGrArUrArUrUrUrArUrArArAAT	rArUrUrUrUrArUrArArArUrArUrCrArUrGrUrArArGrGrArArCrUrG
*siCCR2b	rUrUrGrCrUrArArArCrGrUrCrUrCrUrGrCrArArArCrArGrUrG	rCrArCrUrGrUrUrUrGrCrArGrArGrArCrGrUrUrUrArGrCrArArUrG
*siCCR2b_Mod (modified)	/5Phos/mUmUrGmCrUmArArArCrGrUmCrUmCrUmGrCmArArArCrArGmUG	rCrAmCrUrGrUrUrUmGrCmArGmArGrArCrGrUrUrUrArGmCrAmAmUmG
*Negative control	/5Phos/rCrGrUrUrArArUrCrGrCrGrUrArUrArArUrArCrGrCrGrUdAdT	rArUrArCrGrCrGrUrArUrUrArUrArCrGrCrGrArUrUrArArCrGrArC
*Negative control modified	/5Phos/mCmGrUmUrAmArUrCrGrCrGmUrAmUrAmArUmArCrGrCrGmUdAdT	rArUmArCrGrCrGrUmArUmUrAmUrArCrGrCrGrArUrUrAmArCmGmAmC
*Selected sequence	r: Ribose d: Deoxyribose<br>m: 2'-O-Methyl ribose /5Phos/: Phosphoryl-group	

Supplementary Table 5. Flow cytometry antibodies myeloid panel LAMP1 silencing studies.

Target	Fluorochrome	Type	Isotype species	Isotype Ig	Isotype fragment	Clone	Immunogen	Company	Catalog number	Lot/batch number
**CD115 (CSF-1R)**	BV421	Monoclonal	Rat	IgG2a	κ	AFS98	-	Biolegend	135513	B334938
**Ly6C**	FITC	Monoclonal	Rat	IgG2c	κ	HK1.4	L3 cloned CTL cells	Biolegend	128006	B336286
**Ly6G**	APC-Cy7	Monoclonal	Rat	IgG2a	κ	1A8	Ly-6G transfected EL-4J cell line	Biolegend	127624	B348489
**CD11b**	BV785	Monoclonal	Rat	IgG2b	κ	M1/70	C57BL/10 splenocytes	Biolegend	101243	B373879
**CD45**	PerCP	Monoclonal	Rat	IgG2b	κ	30-F11	Mouse thymus or spleen	Biolegend	103130	B349380/ B343481
**CD11c**	PE-Cy7	Monoclonal	Armenian Hamster	IgG		N418	Mouse spleen dendritic cells	Biolegend	117318	B363386/B162483)
**F4/80**	PE	Monoclonal	Rat	IgG2a	κ	BM8	Murine macrophages	Biolegend	123110	B374509
**LAMP1 (CD107a)**	APC	Monoclonal	Rat	IgG2a	κ	1D4B	NIH/3T3 mouse embryonic fibroblast tissue culture cell membranes	Biolegend	121614	B338818

Supplementary Table 6. Flow cytometry antibodies progenitor panel LAMP1 silencing studies.

Target	Fluorochrome	Type	Isotype species	Isotype Ig	Isotype fragment	Clone	Immunogen	Company	Catalog number	Lot/batch number
CD16/32	BV510	Monoclonal	Rat	IgG2a	λ	93	Sorted pre-B cells	Biolegend	101333	B336556
CD48	APC-Cy7	Monoclonal	Armenian Hamster	IgG		HM48-1	Mouse T lymphoma MBL-2	Biolegend	103432	B329749
CD41	PE-Cy7	Monoclonal	Rat	IgG1	κ	MWReg30	Mouse platelets	Biolegend	133916	B293729
CD135 (Flt3/Flk2)	PerCP-eFluor 710	Monoclonal	Rat	IgG2a	κ	A2F10		ThermoFisher	46-1351-82	2202073
CD150	BV605	Monoclonal	Rat	IgG2a	λ	TC15-12F12.2	Mouse SLAM-human IgG1 fusion protein	Biolegend	115927	B361886
LAMP1 (CD107a)	APC	Monoclonal	Rat	IgG2a	κ	1D4B	NIH/3T3 mouse embryonic fibroblast tissue culture cell membranes	Biolegend	121614	B338818
Lineage cocktail: CD3+CD11b+CD45R/B220+Ly-76+Ly6G+Ly6C	FITC							Biolegend		
CD34	BV421	Monoclonal	Rat	IgG2a	κ	SA376A4	Mouse CD34 transfected cells.	Biolegend	152208	B378790
CD117 (c-kit)	BV785	Monoclonal	Rat	IgG2b	κ	ACK2		Biolegend	135138	B387239
Ly-6A/E (Sca-1)	PE	Monoclonal	Rat	IgG2a	κ	W18174A	EL4 cell	Biolegend	160906	B370192

Supplementary Table 7. Flow cytometry antibodies progenitor panel mCherry expression studies.

Target	Fluorochrome	Type	Isotype species	Isotype Ig	Isotype fragment	Clone	Immunogen	Company	Catalog number	Lot/batch number
CD16/32	BV510	Monoclonal	Rat	IgG2a	λ	93	Sorted pre-B cells	Biolegend	101333	B336556
Lineage cocktail: CD3+CD11b+CD45R/B220+Ly-76+Ly6G+Ly6C	FITC							Biolegend	133302	
CD34	BV421	Monoclonal	Rat	IgG2a	κ	SA376A4	Mouse CD34 transfected cells.	Biolegend	152208	B378790
CD117 (c-kit)	BV785	Monoclonal	Rat	IgG2b	κ	ACK2		Biolegend	135138	B387239
Ly-6A/E (Sca-1)	APC	Monoclonal	Rat	IgG2a	κ	W18174A	EL4 cell	Biolegend	160904	B342242

Supplementary Table 8. Flow cytometry antibodies tumor panel.

Target	Fluorochrome	Type	Isotype species	Isotype Ig	Isotype fragment	Clone	Immunogen	Company	Catalog number	Lot/batch number
CD115 (CSF-1R)	BV421	Monoclonal	Rat	IgG2a	κ	AFS98	-	Biolegend	135513	B334938
Ly6C	FITC	Monoclonal	Rat	IgG2c	κ	HK1.4	L3 cloned CTL cells	Biolegend	128006	B336286
CD68	PerCP-Cy5.5	Monoclonal	Rat	IgG2a	κ	FA-11	Purified Con A receptor glycoproteins from the P815 cell line	Biolegend	137010	B354356
CD11b	BV785	Monoclonal	Rat	IgG2b	κ	M1/70	C57BL/10 splenocytes	Biolegend	101243	B373879
CD206 (MMR)	PE-Cy7	Monoclonal	Rat	IgG2a	κ	C068C2	Recombinant mouse CD206 (MMR)	Biolegend	141720	B412642
F4/80	PE	Monoclonal	Rat	IgG2a	κ	BM8	Murine macrophages	Biolegend	123110	B374509
CD192 (CCR2)	APC	Monoclonal	Rat	IgG2b	κ	SA203G11	Mouse CCR2 transfectants	Biolegend	150628	B409090
