# Supplementary material for: Nature-inspired platform nanotechnology for RNA delivery to myeloid cells and their bone marrow progenitors
Source: Nat Nanotechnol. 2025 Feb 3;20(4):532–42. doi: 10.1038/s41565-024-01847-3 (PMC12014499; doi:10.1038/s41565-024-01847-3)
Supplement: Supplementary file 2 — Reporting Summary [file 41565_2024_1847_MOESM2_ESM.pdf]

nature portfolio

Corresponding author(s): Willem J.M. Mulder (willem.mulder@radboudumc.nl) Roy van der Meel (r.v.d.meel@tue.nl)

Last updated by author(s): Nov 12, 2024

Reporting Summary

Nature Portfolio wishes to improve the reproducibility of the work that we publish. This form provides structure for consistency and transparency in reporting. For further information on Nature Portfolio policies, see our Editorial Policies and the Editorial Policy Checklist.

Statistics

For all statistical analyses, confirm that the following items are present in the figure legend, table legend, main text, or Methods section.

n/a	Confirmed	
[ ]	[x]	The exact sample size (*n*) for each experimental group/condition, given as a discrete number and unit of measurement
[ ]	[x]	A statement on whether measurements were taken from distinct samples or whether the same sample was measured repeatedly
[ ]	[x]	The statistical test(s) used AND whether they are one- or two-sided<br>*Only common tests should be described solely by name; describe more complex techniques in the Methods section.*
[ ]	[x]	A description of all covariates tested
[ ]	[x]	A description of any assumptions or corrections, such as tests of normality and adjustment for multiple comparisons
[ ]	[x]	A full description of the statistical parameters including central tendency (e.g. means) or other basic estimates (e.g. regression coefficient) AND variation (e.g. standard deviation) or associated estimates of uncertainty (e.g. confidence intervals)
[ ]	[x]	For null hypothesis testing, the test statistic (e.g. *F*, *t*, *r*) with confidence intervals, effect sizes, degrees of freedom and *P* value noted<br>*Give P values as exact values whenever suitable.*
[x]	[ ]	For Bayesian analysis, information on the choice of priors and Markov chain Monte Carlo settings
[x]	[ ]	For hierarchical and complex designs, identification of the appropriate level for tests and full reporting of outcomes
[x]	[ ]	Estimates of effect sizes (e.g. Cohen's *d*, Pearson's *r*), indicating how they were calculated

Our web collection on statistics for biologists contains articles on many of the points above.

Software and code

Policy information about availability of computer code

Data collection	N/A
Data analysis	N/A

For manuscripts utilizing custom algorithms or software that are central to the research but not yet described in published literature, software must be made available to editors and reviewers. We strongly encourage code deposition in a community repository (e.g. GitHub). See the Nature Portfolio guidelines for submitting code & software for further information.

Data

Policy information about availability of data

All manuscripts must include a data availability statement. This statement should provide the following information, where applicable:

- Accession codes, unique identifiers, or web links for publicly available datasets
- A description of any restrictions on data availability
- For clinical datasets or third party data, please ensure that the statement adheres to our policy

The data supporting the results in this study are available within the main text, the Extended Data, and the Supplementary Information. The source data of the cryo-EM analysis and liver histology are available for research purposes from the corresponding authors upon reasonable request.

Research involving human participants, their data, or biological material

Policy information about studies with human participants or human data. See also policy information about sex, gender (identity/presentation), and sexual orientation and race, ethnicity and racism.

Reporting on sex and gender	N/A
Reporting on race, ethnicity, or other socially relevant groupings	N/A
Population characteristics	N/A
Recruitment	N/A
Ethics oversight	N/A

Note that full information on the approval of the study protocol must also be provided in the manuscript.

Field-specific reporting

Please select the one below that is the best fit for your research. If you are not sure, read the appropriate sections before making your selection.

☒ Life sciences ☐ Behavioural & social sciences ☐ Ecological, evolutionary & environmental sciences

For a reference copy of the document with all sections, see nature.com/documents/nr-reporting-summary-flat.pdf

Life sciences study design

All studies must disclose on these points even when the disclosure is negative.

Sample size	Mimimum group sizes were determined by statistical power calculations (Piface) while keeping rates for type I and II errors <5% and a power of 80%.
Data exclusions	No data points were excluded from the analyses.
Replication	All experiments in this study were replicated, except the in vivo library screening and the tumor experiments.
Randomization	Randomization was always performed. Regarding the tumor experiments: treatment group allocation based on tumor size was performed randomly by dividing mice randomly between cages.
Blinding	Blinding was always implemented. Regarding the tumor experiments: Researcher 1 was responsible for treatment injections, while researcher 2 was responsible for monitoring tumor sizes. This ensures that researcher 2 measures tumor volumes without knowledge of the treatment group assignments.

Reporting for specific materials, systems and methods

We require information from authors about some types of materials, experimental systems and methods used in many studies. Here, indicate whether each material, system or method listed is relevant to your study. If you are not sure if a list item applies to your research, read the appropriate section before selecting a response.

Materials & experimental systems

n/a	Involved in the study	
☐	☒	Antibodies
☐	☒	Eukaryotic cell lines
☒	☐	Palaeontology and archaeology
☐	☒	Animals and other organisms
☒	☐	Clinical data
☒	☐	Dual use research of concern
☒	☐	Plants

Methods

n/a	Involved in the study	
☒	☐	ChIP-seq
☐	☒	Flow cytometry
☒	☐	MRI-based neuroimaging

Antibodies

Antibodies used	All antibodies used for flow cytometry studies are provided in Supplementary Table 5-8.
Validation	Validation statements are available from the manufacturer. Antibody catalog and batch number are provided in the Supplementary

Table 5-8.

Eukaryotic cell lines

Policy information about cell lines and Sex and Gender in Research

Cell line source(s)	RAW264.7 murine macrophages were obtained from ATCC (TIB-71).
Authentication	The cell line was not authenticated .
Mycoplasma contamination	The cell line tested negatively for mycoplasma contamination.
Commonly misidentified lines (See ICLAC register)	No commonly misidentified lines were used in this study.

Animals and other research organisms

Policy information about studies involving animals; ARRIVE guidelines recommended for reporting animal research, and Sex and Gender in Research

Laboratory animals	8-12 weeks old female C57BL/6J mice (Mus musculus) obtained from Charles River Laboratories Germany.
Wild animals	The study did not involve wild animals.
Reporting on sex	The study was conducted with female mice.
Field-collected samples	The study did not involve field-collected samples.
Ethics oversight	All animal experiments were conducted in compliance with European and Dutch guidelines according to the care and use of laboratory mice after approval by the Radboud University Medical Center's Dierexperimentencommissie (DEC) (CCD License: AVD10300 2021 15550).

Note that full information on the approval of the study protocol must also be provided in the manuscript.

Plants

Seed stocks	N/A
Novel plant genotypes	N/A
Authentication	N/A

Flow Cytometry

Plots

Confirm that:

- [ ] The axis labels state the marker and fluorochrome used (e.g. CD4-FITC).
- [ ] The axis scales are clearly visible. Include numbers along axes only for bottom left plot of group (a 'group' is an analysis of identical markers).
- [ ] All plots are contour plots with outliers or pseudocolor plots.
- [ ] A numerical value for number of cells or percentage (with statistics) is provided.

Methodology

Sample preparation	Sample preparation is specified in the Methods section.
Instrument	21-color CytoFLEX LX (Beckman Coulter).
Software	FlowJo.
Cell population abundance	Sorting was not performed.

Gating strategy	Gating Strategy is presented in Supplementary Figure 4.

☒ Tick this box to confirm that a figure exemplifying the gating strategy is provided in the Supplementary Information.
